# A classification approach for genotyping viral sequences based on multidimensional scaling and linear discriminant analysis

**DOI:** 10.1186/1471-2105-11-434

**Published:** 2010-08-21

**Authors:** Jiwoong Kim, Yongju Ahn, Kichan Lee, Sung Hee Park, Sangsoo Kim

**Affiliations:** 1 Department of Bioinformatics & Life Sciences, Soongsil University, Seoul, 156-743, Korea; 2 Current Address: Equispharm Co., Ltd, Suwon, 443-766, Korea; 3 Current Address: Macrogen Inc., Seoul, 153-023, Korea

## Abstract

**Background:**

Accurate classification into genotypes is critical in understanding evolution of divergent viruses. Here we report a new approach, MuLDAS, which classifies a query sequence based on the statistical genotype models learned from the known sequences. Thus, MuLDAS utilizes full spectra of well characterized sequences as references, typically of an order of hundreds, in order to estimate the significance of each genotype assignment.

**Results:**

MuLDAS starts by aligning the query sequence to the reference multiple sequence alignment and calculating the subsequent distance matrix among the sequences. They are then mapped to a principal coordinate space by multidimensional scaling, and the coordinates of the reference sequences are used as features in developing linear discriminant models that partition the space by genotype. The genotype of the query is then given as the maximum *a posteriori *estimate. MuLDAS tests the model confidence by leave-one-out cross-validation and also provides some heuristics for the detection of 'outlier' sequences that fall far outside or in-between genotype clusters. We have tested our method by classifying HIV-1 and HCV nucleotide sequences downloaded from NCBI GenBank, achieving the overall concordance rates of 99.3% and 96.6%, respectively, with the benchmark test dataset retrieved from the respective databases of Los Alamos National Laboratory.

**Conclusions:**

The highly accurate genotype assignment coupled with several measures for evaluating the results makes MuLDAS useful in analyzing the sequences of rapidly evolving viruses such as HIV-1 and HCV. A web-based genotype prediction server is available at http://www.muldas.org/MuLDAS/.

## Background

We are observing rapid growth in the number of viral sequences in the public databases [1]: for example, HIV-1 and HCV sequence entries in NCBI GenBank have doubled almost every three years. These viruses also show great genotypic diversities and thus have been classified into groups, so-called genotypes and subtypes [2,3]. Consequently classifying these virus strains into genotypes or subtypes based on their sequence similarities has become one of the most basic steps in understanding their evolution, epidemiology and developing antiviral therapies or vaccines. The conventional classification methods include the following: (1) the nearest neighbour methods that look for the best match of the query to the representatives of each genotype, so-called references (e.g., [4]); (2) the phylogenetic methods that look for the monophyletic group to which the query branches (e.g., [5]). Since the genotypes have been defined originally as separately clustered groups, these intuitively sound methods have been widely used and quite successful for many cases.

However, with increasing numbers of sequences, we are observing outliers that cannot be clearly classified (e.g., [6]) or for which these methods do not agree. A recent report that compared these different automatic methods with HIV-1 sequences showed less than 50% agreement among them except for subtypes B and C [7]. One of the reasons for the disagreement was attributed to the increasing divergence and complexity caused by recombination. It was also noted that closely related subtypes (B and D) or the subtypes sharing common origin (A and CRF01_AE) showed poor concordance rate among those methods. We think what lies at the bottom of this problem is that the number of reference sequences per subtype was too small; these methods have used two to four reference sequences. Having been carefully chosen by experts among the high-quality whole-genome sequences, they are to cover the diversity of each subtype as much as possible [2]. However with intrinsically small numbers of references per subtype, they cannot address the confidence of subtype predictions; a low E-value of a pairwise alignment or a high bootstrap value of a phylogenetic tree indicates the reliability of the unit operation, but does not necessarily guarantee a confident classification.

Recognition of this issue of lacking a statistical confidence measure, brought about the introduction of the probabilistic methods based on either position-specific scoring matrix [8] or jumping Hidden Markov Models (jpHMM) [9-11] built from multiple sequence alignment (MSA) of each genotype. By using full spectra of reference sequences, jpHMM was effective in detecting recombination breakpoints. Recently, new classification methods based on nucleotide composition strings have been introduced [12]. It is unique in that it bypasses the multiple sequence alignment and still achieves high accuracy. However, it uses only 42 reference sequences and has been tested with 1,156 sequences. Considering the explosive increase in the numbers of these viral sequences, the test cases of these conventional methods were rather small, an order of ten thousands at most. It would be desirable to measure the performance of a new classification method over all the sequences publicly available.

**Figure 1:**
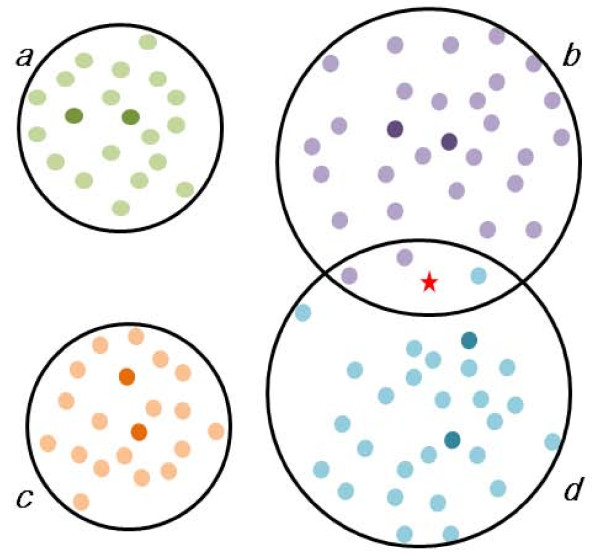
**A schematic diagram illustrating the concept of classification of a viral sequence**. The filled spheres represent known sequences that have been clustered into four groups, ***a ***through ***d***, the boundaries of which are depicted by black circles. Suppose the dark spheres in each cluster represent the respective reference sequences and the red asterisk denotes a query sequence. Since the query is located at the interface of ***b ***and ***d ***clusters, its genotype (or subtype) is elusive. On the other hand, a nearest neighbour method may assign it to the nearest reference sequence, which happens to be ***d ***in this example. If the classification method does not take into account the clustering patterns of the known sequences and relies on the distances to the nearest reference sequences, its result may not be robust to the choice of references.

It is critical to evaluate how well each genotype population is clustered, before attempting to classify a query sequence. Consider a case where the reference sequences are mostly well segregated by genotype except for two or more genotypes that overlap at least partially (see Figure 1 for an illustration); those methods that rely on a few references may not notice this problem and may assign an apparent genotype with a high score. Due to varying mutation rate along the sequence range, the phylogenetic power of each gene segment may also vary [13]. This is particularly critical for relatively short partial sequences. In other words, even the well characterized references that are otherwise distinctively clustered may not be resolved if only part of the sequence region is considered in the classification. The nearest neighbour methods do not evaluate this validity of the background classification models, since they concern the alignments of only query-to-reference, not reference-to-reference. REGA, one of the tree-based methods, concerns whether the query is inside or outside the cluster formed by a group of references [5]. The branching index has been proposed to quantify this and has been useful in detecting outlier sequences [14,15]. A statistical method, jpHMM, reports the posterior probabilities of the subtypes at each query sequence position; based on these, some heuristics is given to assess the uncertainty in detecting recombination region [11].

Here we present a new method, MuLDAS, which develops the background classification models based on the distances among the reference sequences, re-evaluates their validity for each query, and reports the statistical significance of genotype assignment in terms of posterior probabilities. As such, it is suited for the cases where many reference sequences are available. MuLDAS achieves such goals by combining principal coordinate analysis (PCoA) [16] with linear discriminant analysis (LDA), both of which are well established statistical tools with popular usages in biological sciences. PCoA, also known as classical multidimensional scaling (MDS), maps the sequences to a high-dimensional principal coordinate space, while trying to preserve the distance relationships among them as much as possible. It has been widely applied to the discovery of global trends in a sequence set, complementing tree-based methods in phylogenetic analysis [17,18]. Since genotypes have been defined as distinct monophyletic groups in a phylogenetic tree, each genotype should form a well separated cluster in a MDS space if an appropriately high dimension is chosen. In such cases, we can find a set of hyperplanes that separate these clusters and classify a query relative to the hyperplanes. For this purpose, MuLDAS applies LDA [19], a straightforward and powerful classification method, to the MDS coordinates and assigns a query to the genotype that shows the highest posterior probability of membership. This probability can be useful in detecting any ambiguous cases, for which careful examination is required. MuLDAS tests the LDA models through the leave-one-out cross-validation (LOOCV), which can be used to assess the model validity by examining the misclassification rate. As the sequences are represented by coordinates, a simple measure can be also developed for detecting genotype outliers. We have tested the algorithm with virtually all the HIV-1 and HCV sequences available from NCBI GenBank and the results are presented.

## Methods

### Overall Process

**Figure 2:**
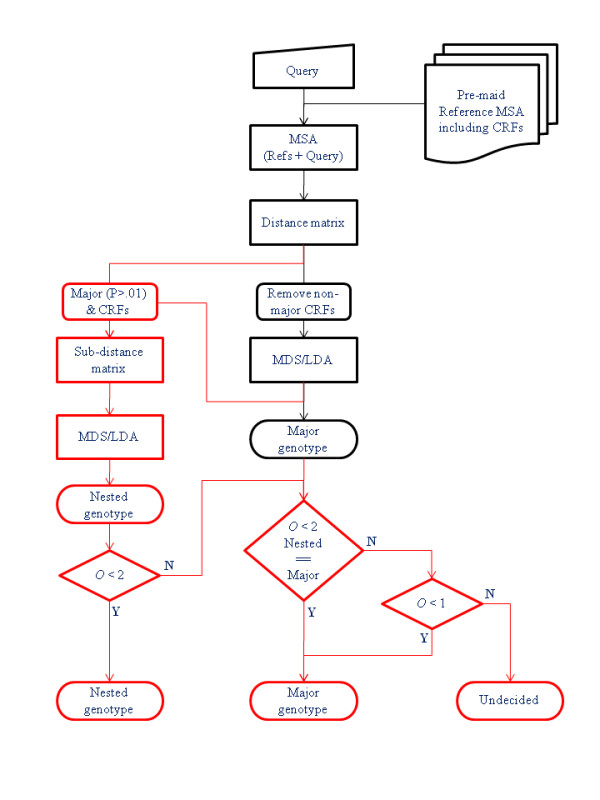
**A flowchart of the algorithm for a given gene segment**. MuLDAS starts by aligning the query with the pre-maid MSA of reference sequences, which includes CRFs in HIV-1. Through this, the gene segments to which the query maps are identified and the whole process is repeated over these gene segments. After distance matrix is obtained, MDS and LDA are performed to classify the query. In HIV-1, only the *major *groups are used in this step. The genotype gives rise to the best posterior probability is reported as the *major *genotype. If nested analysis is not required as for HCV, the process stops here. Otherwise as for HIV-1, an additional process called nested analysis (shown in red) is performed. For the *major *analysis, the genotypes that give rise to P > 0.01 and their associated CRFs are identified, and the subset of the distance matrix corresponding to these genotypes is excised from the original matrix saved in the *major *analysis. After MDS and LDA, the best genotype is reported as the *nested *genotype. Once both *nested *and *major *genotypes are determined, a decision process outlined at the bottom proceeds from left to right and suggests the final outcome (see "a proposed process for subtype decision" section for details).

A flowchart of the algorithm is shown in Figure 2. MuLDAS starts the process by creating a multiple sequence alignment (MSA) of the query with the reference sequences. MuLDAS requires a large number of references, which should be of high quality and with carefully assigned genotypes. Los Alamos National Laboratory (LANL) databases distribute such MSAs of HIV-1 http://www.hiv.lanl.gov/ and HCV http://hcv.lanl.gov/ sequences. LANL also provides the genotype information on each sequence in the MSA. A total of 3,591 nucleotide sequences were included in the 2007 release of HIV-1 MSAs (Supplementary Table 1 in Additional File 1), while a total of 3,093 nucleotide sequences were in HCV MSAs (Supplementary Table 2 in Additional File 1). It should be noted that for some genotypes, more than 100 sequences were found in the MSA, while there were rare genotypes for which only a few reference sequences were included [20,21]. This imbalance in sample sizes is a serious problem to MuLDAS but we propose rather a heuristic solution that is based on the global variance (*vide infra*). For a fair comparison with other methods, we decided to honour the MSA of reference sequences already available from the public databases by aligning the query to this reference MSA, rather than creating MSA by ourselves. This has the advantage of saving the execution time, which is crucial for a web server application (see the section 'Web server development'). The suit of programs, *hmmbuild*, *hmmcalibrate*, and *hmmalign *http://hmmer.janelia.org/ are used for this step. After removing indels in the MSA using a PERL script, the pairwise distance matrix among these sequences is calculated using *distmat *of EMBOSS package http://emboss.sourceforge.net/ with the Jukes-Cantor correction.

**Figure 3:**
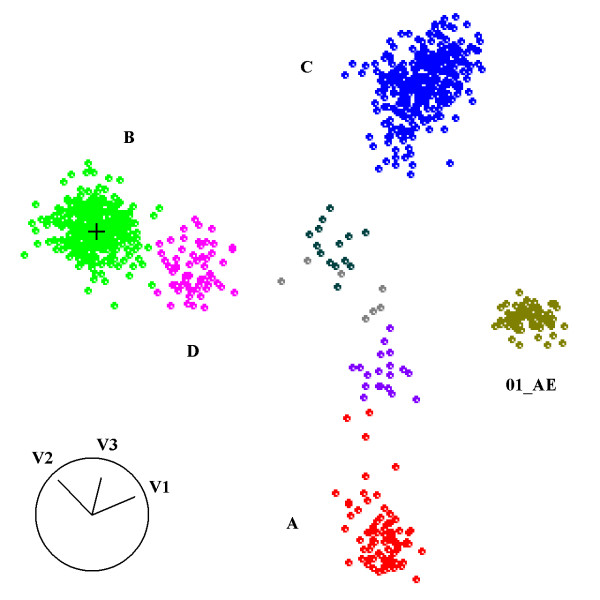
**An exemplary MDS plot of HIV-1 sequences along the first (V1), second (V2), and third (V3) principal coordinate axes**. The reference sequences were shown as small circles colour-coded according to their subtypes. For clarity the subtypes F-K were not labelled. The query was located in the middle of subtype B ('+'). The image was created with *GGobi *http://www.ggobi.org/.

The next step is so-called principal coordinate analysis (PCoA), which turns the distance matrix to a matrix whose components are equivalent to the inner products of the sought coordinates. Through singular value decomposition of the resulting matrix, a set of eigenvectors and associated eigenvalues are obtained up to the specified lower dimensions. The multidimensional coordinates of the sequences whose pairwise Euclidean distances approximate the original distances, are then recovered from a simple matrix operation involving the eigenvectors and eigenvalues (for details see [16]). Each eigenvalue is the amount of variance captured along the axis defined by the corresponding eigenvector, also called as the principal coordinate (PC). For convenience the eigenvalues are sorted in descending order and dimensionality reduction is achieved by taking the top few components. If the within-group variation is negligible, the number of top PCs or the MDS dimensionality, *k*, should be at most *N*-1, where *N *is the number of reference groups. However, depending on the sequence region considered, a genotype might show a complex clustering pattern, splitting into more than one cluster. Consequently we took an empirical approach that surveyed the cross-validation error of the reference sequences for *k *ranging from 1 to 50 (see the next subsection). This step is implemented with *cmdscale *in the R statistical system http://www.r-project.org/. See Figure 3 for an exemplary plot of the MDS result.

The last step of MuLDAS is to develop the discriminant models that best classify the references according to their genotypes and assign the genotype membership to the query according to the models. Here one can envisage applying various classification methods such as K-Nearest Neighbour (K-NN), Support Vector Machine (SVM), and linear classifiers, among others. If the references are well clustered according to their genotype membership, then the simplest methods such as linear discriminant analysis (LDA) or quadratic discriminant analysis (QDA) should work. Both of them work by fitting a Gaussian distribution function to each group centre, while the difference between them is whether global (LDA) or group (QDA) covariance is used. Since it can be expected that the within-group divergences may differ from one group to another, QDA may be better suited. However, the sample size imbalance issue mentioned above prevents applying QDA as it becomes unstable with a small number of references for some genotypes. On the other hand, LDA applies the global covariance commonly to all the genotypes and thus may be more robust to this issue. Although it is not as rigorous as QDA, this heuristic approach works reasonably well as long as the group divergences are not too different from one to another. Once the linear discriminants are calculated based on the reference sequences, the posterior probability of belonging to a particular group is given as a function of so-called Mahalanobis distance from the query to the group centre [19]. To the query, the maximum *a posteriori *(MAP) estimate, that is, the genotype having the maximum probability is then assigned. The posterior probability is scaled by the prior that is proportional to the number of references for each genotype. This step is implemented with *lda *of MASS package in the R statistical system http://www.r-project.org/.

### Cross-Validation of the Prediction Models

The validity of the linear discriminant models are assessed by LOOCV of the genotype membership of the reference sequences. For each one of the references, its genotype is predicted by the models generated from the rest of the references. The misclassification error rate, which is the ratio of the number of misclassified references to the total number of references participated in the validation, is a sensitive measure of the background classification power. Many viral sequences in the public databases are not of the whole genome but cover only a few genes or a part of a gene, and thus their phylogenetic signal may be variable [13]. Consequently we re-evaluate the classification power of each prediction using LOOCV. If the reference sequences are not well resolved in the MDS space for a given query, it would be evident in LOOCV, resulting in a high misclassification rate.

### Outlier Detection

Even if the references are well separated by genotype with a low LOOCV error rate, it might be possible that the query sequence itself is abnormal: it could be a composite of two or more genotypes, located in the middle of several genotypes (a recombinant case); it might be close to only one genotype cluster (having a posterior *P *value close to 1 for that subtype) but far outside the cluster periphery (a divergent case). In the field of multivariate analysis, it is customary to detect outliers by calculating Mahalanobis distance from the sample centre and by comparing it with a chi-square distribution [22]. As the Mahalanobis distances have already been incorporated into the calculation of the LDA posterior probability, we propose a measure somewhat distinct, namely, outlierness, *O*, which is the Euclidian distance from the query to the cluster centre relative to the maximum divergence of the references belonging to that subtype along that direction:

(1) $$O=\frac{{\left|X_{Q}-X_{C}\right|}^{2}}{\underset{R\in S}{\max}\left[\left(X_{R}-X_{C})\cdot (X_{Q}-X_{C}\right)\right]}$$

where *X*_*Q*_, *X*_*R*_, and *X*_*C *_are the MDS vectors of the query, one of the references, and the centre of the reference group, *S*, respectively. The group, *S*, contains all the reference sequences belonging to the genotype to which the query has been classified. If *O *is smaller than 1.0, the query is well inside the cluster, and outside otherwise. We can develop a simple heuristic filter based on this: for example, a threshold can be set at 2.0 in order to tolerate some divergence. A similar measure, the branching index, has been devised for tree-based methods to detect outlier sequences by measuring the relative distance from the node of the query to the most recent common ancestor (MRCA) of the genotype cluster [14,15]. See Supplementary Note 1 in Additional File 2 for the comparison of 'outlierness' with the branching index. If a truly new genotype is emerging, MuLDAS may classify such sequences into one of the genotypes (a nominal genotype). Their posterior probabilities may be very high but the 'outlierness' values from the nominal group would be also very high. We simulated such a situation by leaving all the reference sequences of a given genotype out and classifying them based on the reference sequences from the other groups only. Indeed *O *values were consistently large. See Supplementary Note 2 in Additional File 2 for details.

### Nested Analysis for Recombinant Detection

There are a number of methods for characterizing recombinant viral strains [23]. Similar to the tree-based bootscanning method [24], MuLDAS can be run along the sequence in sliding windows to locate the recombination spot. It is applicable to long sequences only and takes too much time to be served practically through web for a tool such as MuLDAS that relies on large sample sizes unless a cluster farm having several hundred CPUs is employed. Rather than attempting to detect *de novo *recombinant forms by performing sliding-window runs, we classify the query to the well defined common recombinant forms by the following approaches: (a) predicting genotypes gene by gene for a query that encompass more than one gene; (b) re-iteration of the analysis in a 'nested' fashion that includes recombinant reference sequences. HIV-1 and HCV contains an order of 10 genes and thus gene by gene analysis of a whole genome sequence may take 10 times longer than a single gene analysis. If different genotypes are assigned with high confidences to different gene segments of a query, it may hint a recombinant case. For some recombinants, the breakpoint may occur in the middle of a gene. In such cases, it is likely that the posterior probability of classification is not dominated by just one genotype but the second or so would have a non-negligible *P *value. We re-iterate the prediction process in a 'nested' fashion by focusing on the genotypes having the *P *value greater than 0.01 and the associated common recombinant genotypes. For example, the references in the 'nested' round of HIV-1 classification would include CRF02_AG group if the *P *value of either A or G group were greater than 0.01. We have implemented this procedure for classifying HIV-1 sequences, for which some common recombinant groups known as circulating recombinant forms (CRFs) have been described [2]. Although recombinant forms have been known for HCV, no formal definitions of common forms are available at the moment [3].

One may argue in favour of an alternative approach where the reference CRF sequences are included into the MSA of the major group sequences and do the classification in a single operation. In multidimensional scaling, both divergent and close sequences are mapped to the same space, the latter are not well resolved. As CRF sequences are often clustered near their ostensible non-recombinant forms, they are not resolved if they are included in the MSA with all the other major group sequences.

### Web Server Development

Apache web servers that accept a nucleotide sequence as a query and predicts the genotype for each gene segment of the query has been developed, one for each of HIV-1 and HCV. These are freely accessible at http://www.muldas.org/MuLDAS/. Each CGI program written in PERL wraps the component programs that have been downloaded from the respective distribution web sites of HMMER, EMBOSS, and R. As the calculation of distance matrix consumes much of the run time, we split the task into several, typically four, computational nodes, each of which calculates parts of the rows in parallel, and the results are integrated by the master node. A typical subtype prediction of a 1000-bp HIV-1 nucleotide sequence takes around 20 seconds on an Intel Xeon CPU Linux box. The web servers report the MAP genotype of the query as well as the posterior *P *for each genotype, the leave-one-out cross-validation result of the prediction models, and the outlier detection result (see Supplementary Figure 1 in Additional File 3 for screenshots). The 3 D plot of the query and the references in the top three PCs are given in PNG format and an XML file describing all the PCs of the query and the references can be downloaded for a subsequent dynamic interactive visualization with *GGobi *http://www.ggobi.org/ (Figure 3). This is particularly useful for visually examining the quality of clustering and for confirming the outlier detection result that may lead to the discernment of potential new types or recombinants. If the number of reference sequences for a particular genotype, the classification by MuLDAS would be suboptimal. In such cases, interactive visualization of the clustering pattern using *GGobi *may also be useful. For HIV-1, the 'nested' analysis as described above is re-iterated and the result is reported as well.

## Results and Discussion

The MuLDAS algorithm was tested with the sequence datasets of HIV-1 and HCV downloaded from NCBI GenBank. The genotype information of nucleotide sequences was retrieved from the LANL website for 158,578 HIV-1 (including 6,203 CRFs) and 40,378 HCV sequences (non-recombinants only) that have not been used as the reference sequences. For some of the sequences, the genotypes/subtypes were given by the original submitters and otherwise they were assigned by LANL. We considered these datasets as 'gold standards' for benchmarking the performance of MuLDAS.

### Genotype/Subtype nomenclatures of the test datasets

HIV-1 sequences are grouped into M (main), N (non-main), U (unclassified) and O (outgroup) groups [2]. Most of the sequences available belong to M group. As N and O groups are quite distant from M group, the subtypes of M group cannot be well resolved in the MDS plot that includes these remote groups. Consequently, we focused on classifying M group sequences into subtypes, A-D, F-H, J, and K. Among M group subtypes, A and F are sometimes further split into sub-subtypes, A1 and A2, and F1 and F2, respectively [2]. However, some new sequences were still being reported at the subtype level in the LANL database. This was the case even to the sequences included in MSA produced by LANL. Resolving sub-subtypes for relatively short sequences using MuLDAS would require a 'nested' analysis using the relevant subtype sequences only. Due to these reasons, we did not attempt to distinguish sub-subtypes and classified them at the subtype level. Different subtypes of the M group sequences may recombine to form a new strain [1]. If these strains were found in more than three epidemiologically independent patients, they are called circulating recombinant forms (CRFs). Among the CRFs, CRF01_AE was formed by recombination of A and now extinct E strains, and constitutes a large family that is distinct from subtype A [2]. We have called the M group and CRF01_AE subtypes as the 'major' subtypes and the MuLDAS run against them as the 'major' analysis. Supplementary Table 3(a) in Additional File 1 lists the breakdown of the statistics by subtypes and gene segments of all the test nucleotide sequences that have been classified to the 'major' groups by LANL. The distribution was far from uniform, representing study biases: sequences belonged to subtypes H, J, and K were rare; especially for auxiliary proteins such as *vif *and *vpr*, non-B strains were too rare to evaluate the classification accuracy.

HCV sequences are now classified as genotypes 1 through 6 and their subtypes are suffixed by a lower case alphabet: for example, 1a, 2k, 6h and so on [3]. The multiple sequence alignments downloaded from the LANL website included only a few sequences per subtype that were to be used as references by MuLDAS, making it difficult to apply MuLDAS at the subtype level. Since these genotypes were roughly equidistant from each other [3], MuLDAS was applied at the genotype level, and all the subtypes from a genotype were lumped together into a group. See Supplementary Tables 4(a) in Additional File 1 for the breakdown of HCV nucleotide sequences, respectively.

### Determination of MDS dimensionality and assessment of model validity

**Figure 4:**
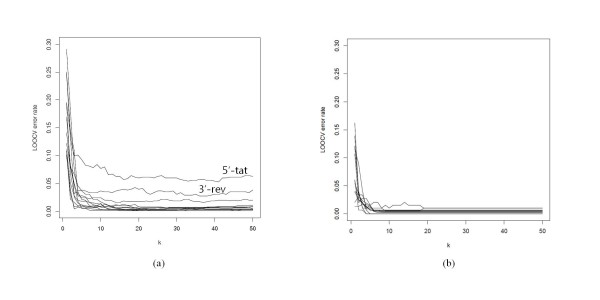
**Surveys of LOOCV error rates by MDS dimensionality, *k*, for each gene segment**. The LOOCV error rates of predicting genotypes (or subtypes) of references sequences were measured by varying the MDS dimensionality, *k*, from 1 through 50 for each gene segment of (a) HIV-1 and (b) HCV nucleotide sequences. Some gene segments showing distinctively higher error rates are labelled. Regardless of sequence types, the error rates reached plateaus after *k *= 10, which was used in the subsequent analyses.

The discriminant models are built solely from the reference sequences and thus their validity is largely irrelevant to the query sequence itself. On the hand, what gene and which portion of the genome the query corresponds to are critical to the discriminatory power as the phylogenetic signal varies along the genome [13,25]. We address this issue by using the LOOCV error rates, which are measured by counting the reference sequences that are misclassified from the class prediction based on the rest of the references. First we looked for the optimum MDS dimensionality, *k*, by surveying the error rates for each whole gene segment. We, then, surveyed the error rates in sliding windows of each gene segment with that *k*. It is expected that the classification power of our discriminant models will increase by representing the sequences in higher and higher *k*. We surveyed the misclassification error rate from LOOCV runs by varying *k *from 1 through 50. As shown in Figure 4, the error rates dropped quickly, reaching a plateau for *k *> = 10. Except for HIV-1 5'-*tat*, excellent performance (error rate < 5%) was observed with *k *> = 10. For HIV-1, short gene segments generally showed poorer performance. While there is no noticeable increase in computational overhead in incrementing *k *from 10 to 50, higher *k *might fall into overfitting. We therefore use *k *= 10 throughout the analysis, while the prediction web server allows changing this parameter.

**Figure 5:**
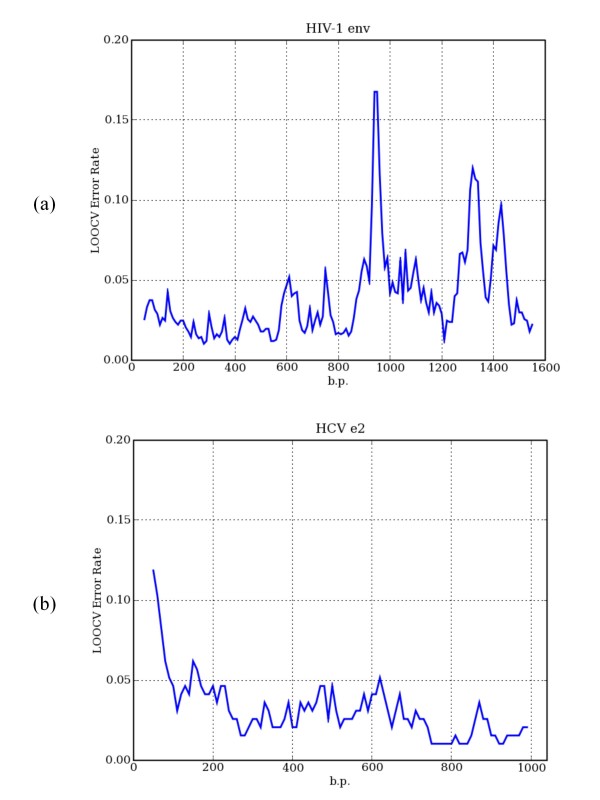
**Representative sliding window plots of LOOCV error rates along gene segments**. The LOOCV error rates were plotted in sliding windows of 100 bp in 10 bp step along the gene segment of (a) HIV-1 *env *and (b) HCV *e2 *nucleotide sequences. The MDS dimensionality was set at *k *= 10 for both cases. Full listings are given in Supplementary Figures 2 and 3 in Additional File 3.

We, then, measured the variation in discriminatory power along the genome or for each gene segment by measuring LOOCV error rates in sliding windows (100 bp windows in 10 bp step). Representative plots for HIV-1 *env *and HCV *e2 *are shown in Figure 5 (see Supplementary Figures 2 and 3 in Additional File 3 for full listings). In general, the error rates were fairly low along the gene segment, although some distinct peaks were observed. The dominant peak seen in HIV-1 *env *and HCV *e2 *corresponds to V3 loop and HVR1, respectively. If the query sequence is composed of primarily of these regions, the high sequence variability is likely to cause suboptimal performance of MuLDAS or any other genotyping tools. In tree-based methods, this will create branches composed of mixed genotypes. In such cases, the assessment of the clustering quality would be ambiguous. On the other hand, MuLDAS provides several means for quality assurance: a LOOCV error rate, posterior probabilities of membership, and an ability to inspect distribution of the sequences in multidimensional space. Even for the cases where the LOOCV error rate is around 10%, the classification can be still valid if the query is found in a region of the multidimensional space where the contaminations by the other genotypes are negligible. Our web-based genotype prediction server masks by default these hyper-variable regions in the query sequence.

### Performance tests

The issue raised above would not be a serious problem as long as the major portion of the query contains good phylogenetic signals. This can be best addressed by running the MuLDAS classification for the entire real world sequences that are not included in the reference panel and tabulating the LOOCV error rates. The shortest query sequence included in our initial benchmark test was 50 bp long. It would be informative to evaluate the performance of MuLDAS with short sequences. Probably the best way to assess this issue is to survey the leave-one-out cross-validation (LOOCV) error rate by sequence length. Since it is purely based on reference sequences only, it would be the optimal performance of MuLDAS. The relevant scatter plots were created for both HIV-1 and HCV nucleotide sequences (see Supplementary Figure 4 in Additional File 3). The LOOCV error rate rises sharply below 100 bp, reaching to about 0.20. Accordingly in the subsequent analysis we used only those sequences longer than 100 bp. Table 1 shows the summary of such runs with all the non-recombinant nucleotide sequences greater than 100 bp. The LOOCV error rates were very low: mean and median being less than 1%. For both HCV and HIV-1 nucleotide sequences, more than 99% of the cases had LOOCV error rates of about 4% or less.

**Table 1:** Summary statistics of the benchmarking results^1^

Virus	**HIV-1**^**2**^	HCV
# of test sequences	153,669	66,488
LOOCV error rate		
mean	0.0100	0.0063
median	0.0068	0.0034
90% percentile	0.0286	0.0155
99% percentile	0.0395	0.0412
MAP^3 ^(% of sequences higher than the cutoff)		
P > = 0.99	97.5	99.6
P > = 0.90	99.1	99.8
P > = 0.50	100.0	100.0
LANL concordance (% of sequences higher than the cutoff)		
All	98.9	96.7
*O *< = 2.0	99.3	96.6

^1^Data as of Jan. 20, 2009.

^2^Major analysis (M-group and CRF01) only.

^3^Maximum *a posteriori*

Having demonstrated that the MuLDAS linear models were well validated, we then surveyed the posterior probability of classification: more than 99% of the cases showed the maximum *a posteriori *probability values of 0.90 or higher, meaning unambiguous calls for most cases (Table 1). The overall concordance rates of the MuLDAS predictions with those retrieved from LANL were 98.9% and 96.7%, respectively for HIV-1 and HCV sequences (Table 1). See the next section for the plausible explanation for the apparently low concordance for HCV.

The concordance rates for each gene and genotype are listed in Tables 2 and 3 for HIV-1 and HCV nucleotide sequences, respectively (see Supplementary Tables 3 and 4 in Additional File 1 for details). If only a few reference sequences are available for any gene-genotype combination, the statistical model of genotype classification for that category would be unreliable: for example, only two to three references were available from each of subtypes H, J, and K (Supplementary Table 1 in Additional File 1). The test sequences in these categories were also extremely rare (Supplementary Tables 3(a) and 4(a) in Additional File 1). Unless more sequences are discovered from these subtypes, their classification using MuLDAS remains to be a challenge.

**Table 2:** The test results with HIV-1 sequences^§^

Category	All		Outlierness < 2.0		No. of reference sequences
		
	Total	% acc.*	Total	% acc.*	
(a) by gene segment					
*Gag*	14,915	97.85	14,570	98.65	1,142
*pol*	64,607	98.67	61,704	99.20	735
*vif*	1,330	99.62	1,324	100.00	945
*vpr*	1,713	99.71	1,702	99.71	810
*tat*	1,987	99.35	1,976	99.44	659
*env*	63,519	99.17	62,652	99.52	1,175
*nef*	5,598	99.59	5,572	99.82	1,559
(b) by subtype					
A	11,907	95.44	11,317	97.74	259
B	112,997	99.53	111,396	99.64	1,750
C	12,785	99.1	12,586	99.52	908
D	3,743	95.99	3,526	98.24	158
F	1,321	96.14	939	97.12	37
G	2,379	94.33	1,800	95.89	65
H	214	71.03	47	51.06	11
J	124	54.84	15	0	3
K	39	69.23	11	54.55	3
01_AE	8,160	98.80	7,863	98.93	152

Total	153,669	98.86	149,500	99.32	3,346

*% accuracy given as 100 × Matched/Total, where Matched is the number cases concordant between MuLDAS and LANL

^§^Major analysis (M-group and CRF01) only.

**Table 3:** The test results with HCV nucleotide sequences

Class	All		Outlierness < 2.0		No. of reference sequences
		
	Total	% acc.*	Total	% acc.*	
(a) by gene segment					
*arfp*	4,333	99.7	4,167	99.78	289
*core*	4,453	99.73	4,412	99.77	463
*e1*	19,353	95.19	19,167	95.15	456
*e2*	16,025	93.32	15,462	93.27	194
*p7*	2,156	100	2,120	100	195
*ns2*	813	100	785	100	166
*ns3*	3,254	99.97	3,125	99.97	154
*ns4a*	950	99.58	796	99.75	253
*ns4b*	921	99.67	787	99.62	148
*ns5a*	5,870	99.98	5,794	99.98	298
*ns5b*	5,115	98.14	4,384	98.02	148
*okamoto*	3,245	97.13	3,240	97.13	2,203
(b) by genotype					
1	49,574	99.15	49,462	99.19	1,709
2	3,544	98.87	3,452	98.99	354
3	6,043	99.7	5,810	99.76	473
4	4,026	66.57	2,875	53.25	346
5	1,182	66.75	641	40.72	72
6	2,119	99.76	1,999	99.9	139

Total	66,488	96.66	64,239	96.61	3,093

*% accuracy given as 100 × Matched/Total, where Matched is the number cases concordant between MuLDAS and LANL

### Outlier filtering

**Figure 6:**
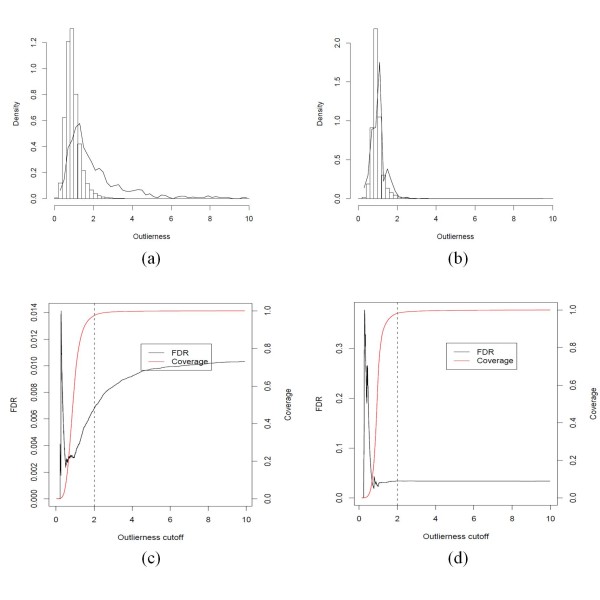
**The density distributions of the outlierness value, *O*, and the corresponding false discovery rates from the benchmark results for HIV-1 and HCV nucleotide sequences**. For all the HIV-1 (a) and HCV (b) sequences used in the benchmark tests (Table 1), the *O *values were surveyed and plotted as the histograms that were separately normalized for the cases concordant with (bar) and discordant to (line) LANL genotypes/subtypes. After filtering out the cases having *O *> cutoff, the discordant ones were counted as false positives. The false positive rates and the proportion of the sequences retained (coverage) were plotted against the *O *cutoff for HIV-1 (c) and HCV (d) sequences. The suggested cutoff is shown by a dashed line. The revised plots after removing the HCV sequences of suspicious genotypes are available in Supplementary Figure 5 in Additional File 3.

Having proposed the outlierness value, *O *(Eq. 1), as an indicator of how well the query clustered with the corresponding references, we examined its distribution: the density plots of *O *showed a sharp peak centred around 1.0 for the concordant predictions (bar), while a long tail up to 10.0 were observed for discordant cases (line) (Figure 6(a, b)). If one treats the discordant cases as false positives, we can survey the false discovery rate (FDR) over the entire range of *O *cutoff (Figure 6(c, d)). It appears that the cutoff at *O *= 2.0 would eliminate much of the misclassifications with a minimal sacrifice of concordant predictions. While noticeable improvement was observed with HIV-1 sequences, no improvement was observed with HCV sequences (Table 1). A closer examination of the confusion table between HCV genotypes before and after the filtering showed some specific patterns of discordance (Supplementary Table 4(e, f) in Additional File 1). It appears they were due to systematic errors in LANL HCV genotype information of these sequences. For example, most of these cases were originated from a few studies that had submitted several hundreds to thousands of sequences. See Additional File 2 Supplementary Note 3 for details. After removing all the sequences from those submissions of suspicious genotype information, the overall concordance rate for HCV were 99.45 and 99.50, respectively before and after applying the cutoff at *O *= 2.0 (Supplementary Table 5 in Additional File 1). Since the concordance was already extremely high, the outlierness filtering showed only marginal improvement. Nevertheless the revised histogram and FDR plots of showed that the cutoff at *O *= 2.0 would eliminate some of the misclassifications (Supplementary Figure 5 in Additional File 3).

### Assessment of the HIV-1 nested analysis results

Many HIV-1 sequences have been described as circulating recombinant forms (CRFs) by LANL. For a total of 9,000 nucleotide gene segments of 8,612 such sequence entries, subtypes were assigned by MuLDAS by the 'nested' analysis (see Methods). After the 'major' analysis of each gene segment, the subtypes having posterior probability greater than 0.01 were identified and the corresponding reference sequences were collected into a pool. The CRF references originated from these subtypes were also added to the pool. The MuLDAS classification model was, then, built based on the pool of references, and was applied to the query sequence. Note that the reference pool was re-collected for each query. A total of 4,994 nucleotide gene segments (derived from 4,949 sequence entries) passed the filtering step (*O *≤ 2.0) and had unambiguous calls (posterior probability ≥ 0.99), with an overall accuracy of 94.67% (Supplementary Table 6 in Additional File 1). It should be noted that the number of reference sequences per gene segment or subtype is not high for CRFs presently and consequently the accuracy reported here should be interpreted carefully. The relatively high accuracy seen with *pol *sequences (Supplementary Tables 6 Additional File 1) are encouraging in that the genes in this segment are the targets of antiviral therapies and recent resistance screenings to help guide treatment regimens frequently sequence these genes [26].

Even with this success, there were still many sequences that failed to pass filtering steps. As a classification tool, MuLDAS has been developed to assign a subtype among a set of known subtypes, and thus not designed to detect a new subtype or recombination pattern. However, MuLDAS may hint some important clues for the analysis of these outlier sequences in terms of outlierness value and a set of posterior probabilities as well as the complex subtype pattern along the sequence. See Supplementary Note 4 in Additional File 2 for the summary of the test runs of MuLDAS with artificial HIV-1 sequences interwoven with two subtypes. The MuLDAS runs displayed complex subtype patterns that were generally congruent with the subtype composition. For the cases where either the recombination spot or subtype composition of the query was substantially different from the common CRFs, its performance were suboptimal. This implies that sliding-window analysis by MuLDAS along the sequence is necessary. We plan to develop MuLDAS further to implement such a feature, exploiting cluster farms with several hundred CPUs.

### A proposed process for subtype decision

It is evident from the previous sections that one has to accept the prediction results if and only if the reported parameters such as posterior probability (*P*) and outlierness (*O*) are reasonable. A working proposal for highly confident genotype assignment may be *P *better than 0.99 and *O *less than 2.0. A straightforward application of such criteria to 100,654 HCV nucleotide gene segment sequences achieved a false positive rate around 2.6%, leaving about 13.9% as undecided (data not shown).

The subtype decision for a HIV-1 sequence is not as straightforward as the genotyping a HCV sequence, as the former has to deal with the issue of recombinant forms. We showed that MuLDAS achieved high classification accuracies with HIV-1 sequence sets that had been pre-segregated as non-recombinants or CRFs. In real situations, we do not know prior to the analysis whether the query is recombinant or not. For HIV-1 sequences, MuLDAS runs a 'major' analysis and subsequently a 'nested' one (see Methods). An automated decision process is, then, needed in order to summarize those statistics in an orderly manner. For example, if the results from the 'major' and 'nested' analyses are different, the user may be confused. Here the objective is to maximize the accuracy without sacrificing the prediction coverage too much. Based on the filtering criteria mentioned above we propose the following strategy: (i) accept the result from 'nested' analysis only if its clustering is tight (*O *≤ 2.0) and the posterior probability is greater than or equal to 0.99; (ii) otherwise, accept the result only if the 'major' and 'nested' analyses agree with each other and one of the outlierness values is less than or equal to 2.0; (iii) or, accept the result from 'major' analysis if its outlierness value is less than or equal to 1.0 and the *P *value is greater than or equal to 0.99. We applied this strategy to 162,669 HIV-1 nucleotide sequences (gene segments), for which the subtype information were available from LANL (Table 4). A total of 130,721 sequences passed the first step with 98.9% accuracy, while the second step (ii) applied to the 31,948 leftovers from the step (i) yielded 22,599 sequences with 94.8% accuracy. The steps (i)-(iii) in this heuristic decision scheme resulted in 98.1% overall prediction accuracy for 94.9% of the total sequences, leaving out 8,274 sequences without subtype assignment (5.1%). By treating the inaccurate ones as false discovery, we can survey the false discovery rate (FDR) over the entire range of *O *value cutoff for each decision step. See Additional File 3 Supplementary Figure 6 for the plots of FDR overlaid with coverage. For the first two steps, *O *cutoff at 2.0 would lower FDR without sacrificing the coverage too much. On the other hand, the last step showed much higher FDR over the entire range. Therefore the cutoff at 1.0, the nominal minimum would be the only choice in this case.

**Table 4:** Accuracy and coverage of each subtype decision step for HIV-1 nucleotide gene segments

Sequence set*	Description	No. of sequences	Accuracy (%)	Coverage (%)
**(1)**	Subtypes given by LANL	162,669		100

**(2)**	[Nested analysis] Outlierness < 2.0 & Pval > 0.99 among (1)	130,721		80.4

	Correctly classified among (2)	129,302	98.91	

**(3)**	(1)-(2)	31,948		19.6

**(4)**	Outlierness < 2.0 & Subtype(major) = subtype(nested) among (3)	22,599		14.1

	Correctly classified among (4)	21,429	94.82	

**(5)**	(3)-(4)	9,349		5.7

**(6)**	[Major analysis] Outlierness < 1.0 & Pval > 0.99 among (5)	1,075		0.7

	Correctly classified among (6)	781	72.65	

**(7)**	(5)-(6)	8,274		5.1

**(8)**	Subtype assigned (2)+(4)+(6)	154,395		94.9

	Correctly classified among (8)	151,512	98.13	

**(9)**	(1)-(8)	8,274		5.1

	Pval < 0.6 among (9)	292		0.2

	Outlierness > 10.0 among (9)	756		0.5

*The sequence sets (2), (4), and (6) correspond to the decision steps (i), (ii), and (iii) in the main text of the "A proposed process for subtype decision" section of Results and Discussion, respectively

While an alternative strategy may maximize the prediction coverage at the loss of the accuracy, our approach minimizes misclassification and leaves the 'twilight zone' to the users' discretion. The latter included some extreme cases such as those in-between multiple subtypes (*P *< 0.6) or far outside the nearest cluster (*O *> 10). The lists constitute about 0.7% of the total HIV-1 nucleotide sequences (Table 4).

### Comparison with other methods

We have validated the performance of MuLDAS in genotyping HCV and subtyping HIV-1 sequences against the benchmark test dataset downloaded from LANL databases. As MuLDAS shows excellent performance, it would be informative to compare with other automatic genotyping (or subtyping) methods. Most published methods report concordance rates with LANL similar to those of MuLDAS, even though one of the tests showed quite discordant results among those methods [7]. However, their test cases were quite limited, not as full scale as those of MuLDAS. It should also be emphasized that all those methods are based on well established core algorithms in the fields of sequence alignment or phylogenetics. As such, appropriate implementations of those methods should work well for the classification of the query, as long as it is well clustered with only one of the genotypes (or subtypes). Therefore it would be more informative to understand the difference of these methods in dealing with a problematic query sequence that is either divergent or recombinant. As there are no such test panels publicly available, we have devised our own panels: one panel of genome sequences (length > 9000 bp) for each of HCV and HIV-1. We downloaded 1,218 and 1,131 such genome sequences from GenBank, respectively for HCV and HIV-1. From LANL, the genotypes were retrieved for 1,116 and 1,086 of them, respectively. MuLDAS ran in the gene-by-gene mode. If all the gene segments of a genome sequence are 'confidently' genotyped by MuLDAS (*O *< 2.0 and *P *> 0.99) and agree with LANL, we count it as a concordant case, otherwise discordant.

For HCV, 1,098 out of 1,116 were concordant, leaving 18 cases as discordant (98.4% accuracy). Since we did not included any recombinant sequences into the reference panels for HCV, all these concordant cases corresponded to 'pure', non-recombinant forms. On the other hand, nine out of 18 discordant cases were designated as recombinant forms by LANL. The gene-by-gene predictions by MuLDAS for these nine sequences were congruent with their recombination patterns. For example, LANL genotype was "1a/2a" for the sequence entry AX057088, while MuLDAS predicted '1' and '2' for six and five segments, respectively. We labelled such cases as "Recombination inferable". Including these 'partial success' cases, the success rate goes up to 99.2% (Table 5). Among the remaining nine cases, EU643835 was designated as a pure genotype '6' by LANL, while MuLDAS displayed '2/6' recombination pattern. NCBI Genotyping Tool and REGA also indicated a similar recombination pattern. The next eight cases were described as non-recombinant forms of genotype 4 by LANL. There was no consensus among the three genotyping methods: many segments were not genotyped 'confidently' by MuLDAS; NCBI Tool reported recombinant forms; REGA reported some as pure forms as LANL. The last one, EF108306, belonged to genotype 7, which has been defined recently and has not been represented in the reference sets, yet. The full listing of the genotype results of these genome sequences is available in Additional File 4.

**Table 5:** Concordance between LANL and MuLDAS in genotypes for the genome sequences longer than 9,000 bp

Sequence set		HCV	HIV-1
(1) Genome sequences downloaded		1,218	1,131
(2) LANL genotypes known in (1)		1,116	1,086
(3) All confidently genotyped gene segments concordant with LANL genotypes		1,098	938
(4) Some confidently genotyped segments discordant to LANL genotypes		18	148
(5) Accuracy [(3)/(2)]%		98.4	86.3
(6) Recombination pattern 'inferable' from MuLDAS results among (4)		9	103

(7) Including 'partial' successes [(3)+(6)]	cases	1,107	1,041
	%	99.2	95.9

For HIV-1, based on the strict criteria mentioned above, 938 out of 1,086 were concordant with LANL, leaving 148 cases as discordant (86.3% accuracy). Since we classify a HIV-1 sequence into M-groups or CRFs (01~16). Any sequences that do not belong to these groups are bound to be discordant in this analysis. Indeed all 148 but seven discordant cases were of complex recombinant ones. The genotype compositions predicted by MuLDAS for 103 such cases were congruent with their recombination patterns designated by LANL. For example, LANL genotype for a sequence entry EU220698 was 'AC', a non-CRF recombination of 'A' and 'C'. Among nine segments, six were of 'C' and three were of 'A' by MuLDAS. Including such 'partial success' cases, the success rate goes up to 95.9% (Table 5).

We validated the results for 148 discordant HIV-1 cases with independent runs of both NCBI Genotyping Tool [4] and REGA [5]. NCBI Genotyping Tool offers an option to choose one from various reference sets. Since some of the genome sequences used in this test are included in more recent reference sets, NCBI Genotyping Tool would immediately recognize them with perfect matches. For fair comparisons, we used so-called "2005 pure and CRFs" as the reference set in the test run. Since NCBI Genotyping Tool does not summarize the sliding window result into a single genotype, we devise our own scheme as follows: for each window the best scoring genotype is reported; among them infrequent ones (<10%) were trimmed. When multiple genotypes are finally reported for a genome sequence, the genotype composition is compared with that of LANL. If they are congruent, we label them 'inferable' (73 cases). This scheme was successfully validated with the 938 sequences for which MuLDAS showed concordance with LANL (Sequence set (3) for HIV-1 in Table 5). On the other hand REGA summarizes the sliding window result and report a single genotype. However among 148 sequences, REGA failed to report summarized genotypes for 107 sequences due to poor bootscanning support values (we label them "Failed QC"). Thus we focused on the comparison with NCBI Tool, which showed 97 and 102 cases (including 'inferable') that were concordant with LANL and MuLDAS, respectively. There were 79 cases for which all three agreed on. The results are summarized in Supplementary Table 7 in Additional File 1, while the full listings of the test results are found in Additional File 5.

**Figure 7:**
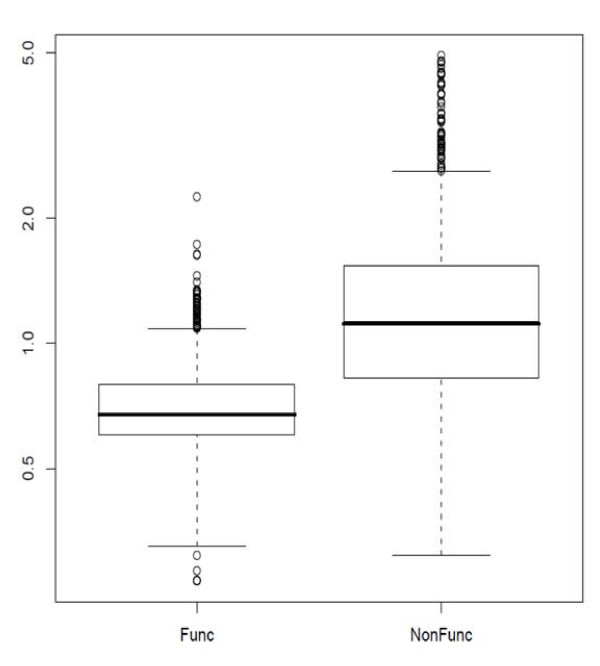
**A boxplot of the outlierness values for HIV-1 hypermutated sequences**. A boxplot of the outlierness value, *O*, was drawn for 561 non-functional and 1,519 functional hypermutated sequences reported by three studies that specifically labelled whether each sequence was 'nonfunctional' or not [27-29].

Hypermutation creates quasi-species that are distant from their ancestors and are often with loss of function in the component genes. It would be interesting to see how MuLDAS behaves with such sequences. There were 14 reports of HIV-1 hypermutations whose sequences had been deposited with the public archives [27-40]. Among 2,308 such sequences, 2,279 were identified from our benchmark results. Since the sequences with non-functional gene components are likely more divergent than the intact sequences, we would compare the degree of divergence between the two groups that have originated from the same studies. Among those 14 reports, three specifically labelled whether the sequence was non-functional or not [27-29]. We measured the outlierness, *O*, parameter for 561 non-functional and 1,519 functional sequences. As shown in Figure 7, the former had distinctively higher *O *values than the latter. This demonstrates that MuLDAS may be useful in pre-screening hypermutation. Here we show an example analysis of a hypermutated sequence with other tools. Janini et al. [27] described a 297 bp HIV-1 *pol *gene coding a non-functional protease due to hypermutation (GenBank accession AY036374.1 and GI: 15192372). The original GenBank record classified it to subtype A. Although NCBI Genotyping Tool also classified it as distinct subtype A, there was no obvious indication of hypermutation. REGA HIV Subtyping Tool assigned it to subtype A with rather high bootstrap (74%), although the topological shape of the phylogenetic tree was unusual. MuLDAS also classified it to subtype A with high confidence (P = 1.0) but more than 10-fold divergent than the known subtype A references (*O_group _*= 10.56). Compared to the maximum radius encompassed by all 735 reference sequences, it was almost four-fold divergent (*O_all _*= 3.99).

## Conclusions

Here we have demonstrated that MuLDAS is a novel approach useful for classifying viral sequences based on a large sample population of reference sequences. As it reports several confidence measures, it is a particularly powerful tool for detecting unusual, problematic sequences that often slip through unnoticed. Explosive growth in number coupled with complex divergence of viral sequences, demands classification tools such as MuLDAS. It has been a while since the previous methods were developed and their performances have not been comprehensively re-evaluated with the sequences emerged since then. MuLDAS achieved remarkable accuracy in the tests that included all HIV-1 or all HCV sequences currently available. As at the core of MuLDAS is MDS of distance matrix followed by LDA, it is conceivable that in place of LDA other classification algorithms such as K-NN or SVM are applied. However, they may not be appropriate as they focus on either a few nearest neighbours (K-NN) or solely on the decision boundary without taking into consideration of the population distribution (SVM). In addition, K-NN may also suffer from the issue of sample size imbalance. MuLDAS algorithm is straightforward enough to be applied to the classification of either nucleotide or peptide sequences. It can be even extended to classify individual subjects into population groups based on a distance matrix of polymorphic markers such as SNP. To sum it up, the approach taken by MuLDAS has far reaching implications for sequence classifications.

## Authors' contributions

JK developed the whole system, did the statistical analysis and helped to draft the manuscript. YA developed the prototype system using HIV-1 nucleotide sequences. KL analyzed the results and helped to draft the manuscript. SHP analyzed the genome sequences and FDR calculations and helped to draft the manuscript. SK conceived of the study, participated in its design, analyzed the results and drafted the manuscript. All authors read and approved the final manuscript.

## Note added in proof

The pre-computed genotype/subtype information is accessible through LinkOut service from NCBI.

## Supplementary Material

Additional file 1 **Supplementary Tables**. Table 1. Number of HIV-1 reference nucleotide sequences per gene segment for each subtype. Table 2. Number of HCV reference nucleotide sequences per gene segment for each genotype. Table 3. Summary statistics of the benchmark test for HIV-1 M group and CRF01_AE nucleotide sequences. Table 4. Summary statistics of the benchmark test for HCV nucleotide sequences. Table 5. Re-analysis of the benchmark test for HCV nucleotide sequences after removing batches of 3,642 sequences that had been submitted by three studies of suspicious genotype information in LANL database (see Additional File 2 Supplementary Note 3 for details). Table 6. Benchmark results of HIV-1 CRF nucleotide sequences from 'nested' analysis. Table 7. Comparison with other methods for 148 cases that were discordant between MuLDAS and LANL for HIV-1 genome sequences longer than 9,000 bp (Sequence set (3) in Table 5) Click here for file

Additional file 2 **Supplementary Notes**. Additional analyses and descriptions provided here include: Note 1. Comparison of 'outlierness' with the branching index in tree-based methods. Note 2. Simulation of new genotype detection by leaving out entire reference sequences of a given genotype and classifying them based on the other reference groups. Note 3. Re-analysis of discordant cases of HCV nucleotide sequences. Note 4. Test results with artificial synthetic HIV-1 nucleotide sequences. Click here for file

Additional file 3 **Supplementary Figures**. Figure 1. Screenshots of MuLDAS web server for subtyping HIV-1 sequences. Figure 2. LOOCV error rates in sliding windows for HIV-1 nucleotide sequences. Figure 3. LOOCV error rates in sliding windows for HCV nucleotide sequences. Figure 4. The scatter plots of LOOCV error rate by sequence length for (a) HIV-1 and (b) HCV nucleotide sequences. Figure 5. The density distributions of the outlierness value, *O*, and the corresponding false discovery rates from the benchmark results for HIV-1 and HCV nucleotide sequences after removing the HCV sequences of suspicious genotypes. Figure 6. The plots of false discovery rates at each step of the proposed process for HIV-1 subtype decision. Click here for file

Additional file 4 **Comparison with other methods for HCV genome sequences**. The following spreadsheets are available: ■ Hcv.summary: summary page to navigate to other sheets. ■ Discordant: the sequences discordant between LANL and MuLDAS. ■ noDiscordant: the sequences concordant between LANL and MuLDAS. Click here for file

Additional file 5 **Comparison with other methods for HIV-1 genome sequences**. The following spreadsheets are available: ■ Hiv1.summary: summary page to navigate to other sheets. ■ Discordant: the sequences discordant between LANL and MuLDAS. ■ noDiscordant: the sequences concordant between LANL and MuLDAS. ■ pivot noDiscord: confusion tables between methods for those sequences in the noDiscord sheet. ■ pivot Discord: confusion tables between methods for those sequences in the Discord sheet. Click here for file
