# Supplementary material for: A classification approach for genotyping viral sequences based on multidimensional scaling and linear discriminant analysis
Source: BMC Bioinformatics. 2010 Aug 21;11:434. doi: 10.1186/1471-2105-11-434 (PMC2936400; doi:10.1186/1471-2105-11-434)
Supplement: Additional file 1 — Supplementary Tables. Table 1. Number of HIV-1 reference nucleotide sequences per gene segment for each subtype. Table 2. Number of HCV reference nucleotide sequences per gene segment for each genotype. Table 3. Summary statistics of the benchmark test for HIV-1 M group and CRF01_AE nucleotide sequences. Table 4. Summary statistics of the benchmark test for HCV nucleotide sequences. Table 5. Re-analysis of the benchmark test for HCV nucleotide sequences after removing batches of 3,642 sequences that had been submitted by three studies of suspicious genotype information in LANL database (see Additional File 2 Supplementary Note 3 for details). Table 6. Benchmark results of HIV-1 CRF nucleotide sequences from 'nested' analysis. Table 7. Comparison with other methods for 148 cases that were discordant between MuLDAS and LANL for HIV-1 genome sequences longer than 9,000 bp (Sequence set (3) in Table 5) [file 1471-2105-11-434-S1.DOC]

**Supplementary Materials submitted to *BMC* *Bioinformatics***

A classification approach for genotyping viral sequences based on multidimensional scaling and linear discriminant analysis

Jiwoong Kim^1^, Yongju Ahn^2^, Kichan Lee, Sung Hee Park and Sangsoo Kim^*^

Department of Bioinformatics & Life Sciences, Soongsil University, Seoul, Korea 156-743.

Present addresses: ^1^Equispharm Co., Ltd, Suwon, Korea 443-766. ^2^Macrogen Inc., Seoul, Korea 153-023

*Contact sskimb@ssu.ac.kr

**Supplementary Table 1.** Number of HIV-1 reference nucleotide sequences per gene segment for each subtype

| **Subtype** | **gag** | **pol** | **vif** | **vpr** | **tat** | **vpu** | **env** | **nef** | **Dist. Acc.** |
| --- | --- | --- | --- | --- | --- | --- | --- | --- | --- |
| (a) M-group and CRF01_AE used in the 'major' analysis | | | | | | | | | |
| **A** | 133 | 59 | 74 | 56 | 63 | 70 | 82 | 109 | 259 |
| **B** | 265 | 168 | 370 | 285 | 159 | 321 | 454 | 766 | 1,750 |
| **C** | 553 | 371 | 364 | 364 | 307 | 397 | 464 | 497 | 908 |
| **D** | 76 | 46 | 48 | 44 | 44 | 54 | 60 | 80 | 158 |
| **F** | 15 | 12 | 13 | 9 | 12 | 12 | 16 | 13 | 37 |
| **G** | 21 | 16 | 17 | 15 | 15 | 22 | 23 | 36 | 65 |
| **H** | 3 | 3 | 3 | 3 | 3 | 6 | 3 | 7 | 11 |
| **J** | 1 | 1 | 1 | 1 | 1 | 2 | 1 | 2 | 3 |
| **K** | 3 | 2 | 2 | 2 | 2 | 2 | 2 | 2 | 3 |
| **01_AE** | 72 | 57 | 53 | 31 | 53 | 46 | 70 | 47 | 152 |
| **Subtotal** | 1,142 | 735 | 945 | 810 | 659 | 932 | 1,175 | 1,559 | 3,346 |
| (b) Other CRFs used in the 'nested' analysis | | | | | | | | | |
| **02_AG** | 59 | 43 | 47 | 43 | 35 | 46 | 55 | 69 | 137 |
| **03_AB** | 1 | 1 | 1 | 1 | 1 | 1 | 1 | 1 | 3 |
| **04_cpx** | 3 | 3 | 3 | 3 | 3 | 3 | 3 | 3 | 3 |
| **05_DF** | 4 | 3 | 3 | 3 | 3 | 2 | 3 | 3 | 4 |
| **06_cpx** | 9 | 5 | 3 | 5 | 6 | 5 | 6 | 11 | 16 |
| **07_BC** | 3 | 1 | 0 | 1 | 1 | 0 | 10 | 1 | 13 |
| **08_BC** | 5 | 1 | 0 | 1 | 1 | 1 | 2 | 1 | 9 |
| **09_cpx** | 5 | 4 | 4 | 4 | 4 | 4 | 4 | 3 | 5 |
| **10_CD** | 3 | 3 | 3 | 3 | 2 | 3 | 3 | 3 | 3 |
| **11_cpx** | 10 | 10 | 9 | 9 | 9 | 9 | 11 | 6 | 12 |
| **12_BF** | 10 | 9 | 9 | 8 | 10 | 12 | 10 | 8 | 13 |
| **13_cpx** | 3 | 3 | 3 | 2 | 3 | 3 | 3 | 3 | 4 |
| **14_BG** | 1 | 1 | 3 | 1 | 2 | 3 | 3 | 14 | 16 |
| **15_01B** | 5 | 3 | 3 | 3 | 2 | 2 | 4 | 4 | 5 |
| **16_A2D** | 2 | 2 | 2 | 2 | 2 | 2 | 2 | 2 | 2 |
| **Subtotal** | 123 | 92 | 93 | 89 | 84 | 96 | 120 | 132 | 245 |
| **Total** | 1,265 | 827 | 1,038 | 899 | 743 | 1,028 | 1,295 | 1,691 | 3,591 |

**Supplementary Table 2.** Number of HCV reference nucleotide sequences per gene segment for each genotype

| **Genotype** | **arfp** | **core** | **e1** | **e2** | **p7** | **ns2** | **ns3** | **ns4a** | **ns4b** | **ns5a** | **ns5b** | **Okam-oto** | **Dist. Acc.** |
| --- | --- | --- | --- | --- | --- | --- | --- | --- | --- | --- | --- | --- | --- |
| **1** | 169 | 292 | 307 | 136 | 148 | 120 | 107 | 192 | 102 | 225 | 101 | 1,085 | 1,709 |
| **2** | 50 | 67 | 57 | 32 | 30 | 29 | 29 | 36 | 29 | 29 | 28 | 268 | 354 |
| **3** | 29 | 38 | 39 | 12 | 6 | 6 | 7 | 13 | 6 | 33 | 6 | 380 | 473 |
| **4** | 7 | 14 | 10 | 1 | 1 | 1 | 1 | 1 | 1 | 1 | 2 | 323 | 346 |
| **5** | 4 | 12 | 8 | 3 | 2 | 2 | 2 | 3 | 2 | 2 | 2 | 55 | 72 |
| **6** | 30 | 40 | 35 | 10 | 8 | 8 | 8 | 8 | 8 | 8 | 9 | 92 | 139 |
| **Total** | 289 | 463 | 456 | 194 | 195 | 166 | 154 | 253 | 148 | 298 | 148 | 2,203 | 3,093 |

**Supplementary Table 3.** Summary statistics of the benchmark test for HIV-1 M group and CRF01_AE nucleotide sequences

(a) Number of gene segments per subtype before filtering

| **Gene** | **A** | **B** | **C** | **D** | **F** | **G** | **H** | **J** | **K** | **01_AE** | **Total** |
| --- | --- | --- | --- | --- | --- | --- | --- | --- | --- | --- | --- |
| **gag** | 2,463 | 8,179 | 2,439 | 672 | 128 | 406 | 35 | 15 | 11 | 567 | 14,915 |
| **pol** | 2,887 | 49,803 | 4,550 | 1,067 | 753 | 1,032 | 75 | 58 | 8 | 4,374 | 64,607 |
| **vif** | 49 | 1,056 | 85 | 27 | 3 | 3 | 0 | 1 | 0 | 106 | 1,330 |
| **vpr** | 41 | 1,502 | 43 | 13 | 4 | 3 | 0 | 1 | 0 | 106 | 1,713 |
| **tat** | 65 | 1,666 | 100 | 42 | 4 | 3 | 0 | 1 | 0 | 106 | 1,987 |
| **env** | 6,233 | 46,171 | 5,058 | 1,850 | 414 | 910 | 101 | 47 | 19 | 2,716 | 63,519 |
| **nef** | 169 | 4,620 | 510 | 72 | 15 | 22 | 3 | 1 | 1 | 185 | 5,598 |
| **Total** | 11,907 | 112,997 | 12,785 | 3,743 | 1,321 | 2,379 | 214 | 124 | 39 | 8,160 | 153,669 |
| **Dist. Acc.** | 11,629 | 110,706 | 12,463 | 3,677 | 1,300 | 2,354 | 214 | 118 | 39 | 7,466 | 149,966 |

(b) Number of gene segments per subtype after outlierness filtering (< 2.0)

| **Gene** | **A** | **B** | **C** | **D** | **F** | **G** | **H** | **J** | **K** | **01_AE** | **Total** |
| --- | --- | --- | --- | --- | --- | --- | --- | --- | --- | --- | --- |
| **gag** | 2,367 | 8,165 | 2,400 | 657 | 91 | 335 | 5 | 0 | 3 | 547 | 14,570 |
| **pol** | 2,587 | 48,299 | 4,438 | 994 | 509 | 730 | 9 | 2 | 0 | 4,136 | 61,704 |
| **vif** | 49 | 1,056 | 85 | 23 | 2 | 3 | 0 | 0 | 0 | 106 | 1,324 |
| **vpr** | 41 | 1,494 | 43 | 13 | 2 | 3 | 0 | 0 | 0 | 106 | 1,702 |
| **tat** | 62 | 1,661 | 100 | 41 | 3 | 3 | 0 | 0 | 0 | 106 | 1,976 |
| **env** | 6,048 | 46,107 | 5,010 | 1,732 | 319 | 706 | 30 | 12 | 7 | 2,681 | 62,652 |
| **nef** | 163 | 4,614 | 510 | 66 | 13 | 20 | 3 | 1 | 1 | 181 | 5,572 |
| **Total** | 11,317 | 111,396 | 12,586 | 3,526 | 939 | 1,800 | 47 | 15 | 11 | 7,863 | 149,500 |
| **Dist. Acc.** | 11,055 | 109,119 | 12,264 | 3,462 | 925 | 1,777 | 47 | 15 | 11 | 7,175 | 145,850 |

(c) Subtype prediction concordance (%) with LANL benchmark test dataset before filtering

| **Gene** | **A** | **B** | **C** | **D** | **F** | **G** | **H** | **J** | **K** | **01_AE** | **Total** |
| --- | --- | --- | --- | --- | --- | --- | --- | --- | --- | --- | --- |
| **gag** | 94.76 | 99.16 | 99.38 | 97.77 | 89.06 | 90.64 | 71.43 | 66.67 | 81.82 | 95.94 | 97.85 |
| **pol** | 88.50 | 99.38 | 99.08 | 96.81 | 98.41 | 96.90 | 96.00 | 56.90 | 75.00 | 98.40 | 98.67 |
| **vif** | 100.0 | 100.0 | 100.0 | 85.19 | 100.0 | 100.0 |  | 0.00 |  | 100.0 | 99.62 |
| **vpr** | 100.0 | 99.80 | 95.35 | 100.0 | 100.0 | 100.0 |  | 100.0 |  | 100.0 | 99.71 |
| **tat** | 84.62 | 100.0 | 100.0 | 95.24 | 100.0 | 100.0 |  | 0.00 |  | 100.0 | 99.35 |
| **env** | 98.91 | 99.69 | 98.89 | 95.14 | 94.44 | 92.97 | 51.49 | 51.06 | 57.89 | 99.85 | 99.17 |
| **nef** | 97.63 | 99.81 | 100.0 | 93.06 | 86.67 | 95.45 | 100.0 | 0.00 | 100.0 | 99.46 | 99.59 |
| **Total** | 95.44 | 99.53 | 99.10 | 95.99 | 96.14 | 94.33 | 71.03 | 54.84 | 69.23 | 98.80 | 98.86 |

(d) Subtype prediction concordance (%) with LANL test dataset after outlierness filtering (< 2.0)

| **Gene** | **A** | **B** | **C** | **D** | **F** | **G** | **H** | **J** | **K** | **01_AE** | **Total** |
| --- | --- | --- | --- | --- | --- | --- | --- | --- | --- | --- | --- |
| **gag** | 97.93 | 99.24 | 99.67 | 98.02 | 95.60 | 89.55 | 60.00 | 0.00 | 66.67 | 95.98 | 98.65 |
| **pol** | 93.54 | 99.56 | 99.55 | 97.89 | 99.21 | 99.04 | 100.0 | 0.00 | 0.00 | 98.57 | 99.20 |
| **vif** | 100.0 | 100.0 | 100.0 | 100.0 | 100.0 | 100.0 | 0.00 | 0.00 | 0.00 | 100.0 | 100.0 |
| **vpr** | 100.0 | 99.80 | 95.35 | 100.0 | 100.0 | 100.0 | 0.00 | 0.00 | 0.00 | 100.0 | 99.71 |
| **tat** | 85.48 | 100.0 | 100.0 | 95.12 | 100.0 | 100.0 | 0.00 | 0.00 | 0.00 | 100.0 | 99.44 |
| **env** | 99.49 | 99.73 | 99.40 | 98.73 | 94.36 | 95.47 | 30.00 | 0.00 | 42.86 | 99.89 | 99.52 |
| **nef** | 100.0 | 99.91 | 100.0 | 93.94 | 92.31 | 100.0 | 100.0 | 0.00 | 100.0 | 100.0 | 99.82 |
| **Total** | 97.74 | 99.64 | 99.52 | 98.24 | 97.12 | 95.89 | 51.06 | 0.00 | 54.55 | 98.93 | 99.32 |

(e) Confusion table (LANL on the left, MuLDAS at the top)

| **LANL** | **A** | **B** | **C** | **D** | **F** | **G** | **H** | **J** | **K** | **01_AE** | **Total** |
| --- | --- | --- | --- | --- | --- | --- | --- | --- | --- | --- | --- |
| **A** | 11,364 | 14 | 16 | 79 | 6 | 77 | 69 | 28 | 8 | 246 | 11,907 |
| **B** | 71 | 112,466 | 45 | 152 | 92 | 22 | 6 | 8 | 9 | 126 | 112,997 |
| **C** | 22 | 30 | 12,670 | 7 | 19 | 4 | 13 | 8 | 11 | 1 | 12,785 |
| **D** | 32 | 24 | 4 | 3,593 | 34 | 2 | 8 | 6 | 39 | 1 | 3,743 |
| **F** | 4 | 14 | 1 | 2 | 1,270 | 7 | 1 | 2 | 19 | 1 | 1,321 |
| **G** | 38 | 33 | 3 | 1 | 14 | 2,244 | 25 | 10 | 9 | 2 | 2,379 |
| **H** | 14 | 0 | 1 | 1 | 13 | 17 | 152 | 15 | 1 | 0 | 214 |
| **J** | 3 | 0 | 1 | 6 | 17 | 6 | 11 | 68 | 11 | 1 | 124 |
| **K** | 0 | 0 | 0 | 0 | 9 | 0 | 3 | 0 | 27 | 0 | 39 |
| **01_AE** | 43 | 48 | 1 | 3 | 1 | 0 | 1 | 0 | 1 | 8,062 | 8,160 |
| **Total** | 11,591 | 112,629 | 12,742 | 3,844 | 1,475 | 2,379 | 289 | 145 | 135 | 8,440 | 153,669 |

(f) Confusion table (LANL on the left, MuLDAS at the top) after outlierness filtering (< 2.0)

| **LANL** | **A** | **B** | **C** | **D** | **F** | **G** | **H** | **J** | **K** | **01_AE** | **Total** |
| --- | --- | --- | --- | --- | --- | --- | --- | --- | --- | --- | --- |
| **A** | 11,061 | 11 | 16 | 66 | 1 | 46 | 0 | 0 | 0 | 116 | 11,317 |
| **B** | 65 | 110,990 | 44 | 120 | 52 | 6 | 0 | 0 | 0 | 119 | 111,396 |
| **C** | 21 | 23 | 12,526 | 3 | 12 | 0 | 0 | 0 | 0 | 1 | 12,586 |
| **D** | 30 | 21 | 3 | 3,464 | 5 | 2 | 1 | 0 | 0 | 0 | 3,526 |
| **F** | 3 | 14 | 1 | 2 | 912 | 5 | 1 | 0 | 1 | 0 | 939 |
| **G** | 30 | 32 | 3 | 0 | 9 | 1,726 | 0 | 0 | 0 | 0 | 1,800 |
| **H** | 12 | 0 | 1 | 1 | 4 | 5 | 24 | 0 | 0 | 0 | 47 |
| **J** | 3 | 0 | 1 | 2 | 6 | 2 | 1 | 0 | 0 | 0 | 15 |
| **K** | 0 | 0 | 0 | 0 | 5 | 0 | 0 | 0 | 6 | 0 | 11 |
| **01_AE** | 39 | 44 | 0 | 1 | 0 | 0 | 0 | 0 | 0 | 7,779 | 7,863 |
| **Total** | 11,264 | 111,135 | 12,595 | 3,659 | 1,006 | 1,792 | 27 | 0 | 7 | 8,015 | 149,500 |

**Supplementary Table 4.** Summary statistics of the benchmark test for HCV nucleotide sequences

(a) Number of gene segments per genotype before filtering

| **gene** | **1** | **2** | **3** | **4** | **5** | **6** | **Total** |
| --- | --- | --- | --- | --- | --- | --- | --- |
| **arfp** | 2,793 | 311 | 554 | 221 | 20 | 434 | 4,333 |
| **core** | 2,869 | 330 | 560 | 238 | 22 | 434 | 4,453 |
| **e1** | 13,814 | 1,249 | 2,092 | 1,550 | 309 | 339 | 19,353 |
| **e2** | 12,677 | 649 | 1,224 | 1,078 | 264 | 133 | 16,025 |
| **p7** | 2,067 | 8 | 16 | 20 | 1 | 44 | 2,156 |
| **ns2** | 720 | 11 | 15 | 20 | 1 | 46 | 813 |
| **ns3** | 2,763 | 65 | 251 | 22 | 107 | 46 | 3,254 |
| **ns4a** | 565 | 16 | 200 | 20 | 108 | 41 | 950 |
| **ns4b** | 700 | 11 | 41 | 20 | 108 | 41 | 921 |
| **ns5a** | 5,487 | 50 | 259 | 20 | 1 | 53 | 5,870 |
| **ns5b** | 3,260 | 453 | 504 | 442 | 122 | 334 | 5,115 |
| **okamoto** | 1,859 | 391 | 327 | 375 | 119 | 174 | 3,245 |
| **Total** | 49,574 | 3,544 | 6,043 | 4,026 | 1,182 | 2,119 | 66,488 |
| **Dist. Acc.** | 30,189 | 2,301 | 4,043 | 2,176 | 556 | 1,113 | 40,378 |

(b) Number of gene segments per genotype after outlierness filtering (< 2.0)

| **gene** | **1** | **2** | **3** | **4** | **5** | **6** | **Total** |
| --- | --- | --- | --- | --- | --- | --- | --- |
| **arfp** | 2,781 | 306 | 551 | 133 | 3 | 393 | 4,167 |
| **core** | 2,868 | 327 | 559 | 229 | 9 | 420 | 4,412 |
| **e1** | 13,808 | 1,243 | 2,091 | 1,438 | 250 | 337 | 19,167 |
| **e2** | 12,654 | 642 | 1,130 | 695 | 212 | 129 | 15,462 |
| **p7** | 2,067 | 8 | 10 | 0 | 1 | 34 | 2,120 |
| **ns2** | 720 | 11 | 15 | 0 | 1 | 38 | 785 |
| **ns3** | 2,761 | 65 | 250 | 0 | 4 | 45 | 3,125 |
| **ns4a** | 565 | 16 | 157 | 1 | 37 | 20 | 796 |
| **ns4b** | 697 | 11 | 35 | 0 | 3 | 41 | 787 |
| **ns5a** | 5,470 | 50 | 220 | 0 | 1 | 53 | 5,794 |
| **ns5b** | 3,212 | 383 | 465 | 4 | 3 | 317 | 4,384 |
| **okamoto** | 1,859 | 390 | 327 | 375 | 117 | 172 | 3,240 |
| **Total** | 49,462 | 3,452 | 5,810 | 2,875 | 641 | 1,999 | 64,239 |
| **Dist. Acc.** | 30,142 | 2,287 | 3,919 | 2,020 | 429 | 1,094 | 39,891 |

(c) Genotype prediction concordance (%) with LANL benchmark test dataset before filtering

| **gene** | **1** | **2** | **3** | **4** | **5** | **6** | **Total** |
| --- | --- | --- | --- | --- | --- | --- | --- |
| **arfp** | 99.86 | 98.07 | 99.82 | 100.00 | 100.00 | 99.54 | 99.70 |
| **core** | 99.86 | 98.18 | 99.82 | 100.00 | 100.00 | 99.77 | 99.73 |
| **e1** | 99.32 | 98.80 | 99.90 | 58.32 | 44.34 | 99.71 | 95.19 |
| **e2** | 98.86 | 98.31 | 99.75 | 35.53 | 17.80 | 100.00 | 93.32 |
| **p7** | 100.00 | 100.00 | 100.00 | 100.00 | 100.00 | 100.00 | 100.00 |
| **ns2** | 100.00 | 100.00 | 100.00 | 100.00 | 100.00 | 100.00 | 100.00 |
| **ns3** | 100.00 | 98.46 | 100.00 | 100.00 | 100.00 | 100.00 | 99.97 |
| **ns4a** | 100.00 | 100.00 | 99.00 | 90.00 | 100.00 | 100.00 | 99.58 |
| **ns4b** | 100.00 | 100.00 | 92.68 | 100.00 | 100.00 | 100.00 | 99.67 |
| **ns5a** | 99.98 | 100.00 | 100.00 | 100.00 | 100.00 | 100.00 | 99.98 |
| **ns5b** | 97.36 | 99.78 | 99.40 | 99.55 | 98.36 | 99.70 | 98.14 |
| **okamoto** | 95.32 | 100.00 | 99.08 | 99.73 | 98.32 | 100.00 | 97.13 |
| **Total** | 99.15 | 98.87 | 99.70 | 66.57 | 66.75 | 99.76 | 96.66 |

(d) Genotype prediction concordance (%) after outlierness filtering (< 2.0)

| **gene** | **1** | **2** | **3** | **4** | **5** | **6** | **Total** |
| --- | --- | --- | --- | --- | --- | --- | --- |
| **arfp** | 99.89 | 98.04 | 100.00 | 100.00 | 100.00 | 100.00 | 99.78 |
| **core** | 99.86 | 98.17 | 100.00 | 100.00 | 100.00 | 100.00 | 99.77 |
| **e1** | 99.32 | 98.79 | 99.90 | 55.08 | 31.20 | 99.70 | 95.15 |
| **e2** | 98.96 | 99.07 | 99.73 | 0.00 | 3.30 | 100.00 | 93.27 |
| **p7** | 100.00 | 100.00 | 100.00 |  | 100.00 | 100.00 | 100.00 |
| **ns2** | 100.00 | 100.00 | 100.00 |  | 100.00 | 100.00 | 100.00 |
| **ns3** | 100.00 | 98.46 | 100.00 |  | 100.00 | 100.00 | 99.97 |
| **ns4a** | 100.00 | 100.00 | 99.36 | 0.00 | 100.00 | 100.00 | 99.75 |
| **ns4b** | 100.00 | 100.00 | 91.43 |  | 100.00 | 100.00 | 99.62 |
| **ns5a** | 99.98 | 100.00 | 100.00 |  | 100.00 | 100.00 | 99.98 |
| **ns5b** | 97.48 | 99.74 | 99.57 | 75.00 | 66.67 | 99.68 | 98.02 |
| **okamoto** | 95.32 | 100.00 | 99.08 | 99.73 | 98.29 | 100.00 | 97.13 |
| **Total** | 99.19 | 98.99 | 99.76 | 53.25 | 40.72 | 99.90 | 96.61 |

(e) Confusion table (LANL on the left, MuLDAS at the top) before filtering

| **LANL** | **1** | **2** | **3** | **4** | **5** | **6** | **Total** |
| --- | --- | --- | --- | --- | --- | --- | --- |
| **1** | 49153 | 356 | 51 | 7 | 3 | 4 | 49574 |
| **2** | 32 | 3504 | 0 | 6 | 0 | 2 | 3544 |
| **3** | 9 | 6 | 6025 | 0 | 1 | 2 | 6043 |
| **4** | 1179 | 0 | 164 | 2680 | 1 | 2 | 4026 |
| **5** | 19 | 7 | 179 | 0 | 789 | 188 | 1182 |
| **6** | 0 | 1 | 1 | 0 | 3 | 2114 | 2119 |
| **Total** | 50392 | 3874 | 6420 | 2693 | 797 | 2312 | 66488 |

(f) Confusion table (LANL on the left, MuLDAS at the top) (outlierness < 2.0)

| **LANL** | **1** | **2** | **3** | **4** | **5** | **6** | **Total** |
| --- | --- | --- | --- | --- | --- | --- | --- |
| **1** | 49,060 | 350 | 47 | 1 | 0 | 4 | 49,462 |
| **2** | 31 | 3,417 | 0 | 2 | 0 | 2 | 3,452 |
| **3** | 9 | 3 | 5,796 | 0 | 0 | 2 | 5,810 |
| **4** | 1,179 | 0 | 164 | 1,531 | 0 | 1 | 2,875 |
| **5** | 14 | 7 | 176 | 0 | 261 | 183 | 641 |
| **6** | 0 | 1 | 1 | 0 | 0 | 1,997 | 1,999 |
| **Total** | 50,293 | 3,778 | 6,184 | 1,534 | 261 | 2,189 | 64,239 |

(g) Re-analyses of the mismatches in (f), for which LANL genotypes were **4** and MuLDAS predicted **1**

| **NCBI genotype** | **REGA genotype** | | | | **Subtotal** |
| --- | --- | --- | --- | --- | --- |
|  | **QC failure** | **1** | **4** | **N.A.** |  |
| **1** | 1 | 564 |  |  | 565 |
| **4** |  |  | 46 |  | 46 |
| **N.A.** |  |  |  | 3 | 3 |
| **Total** | 1 | 564 | 46 | 3 | 614 |

(h) Re-analyses of the mismatches in (f), for which LANL genotypes were **5** and MuLDAS predicted **3**

| **NCBI genotype** | **REGA genotype** | | **Subtotal** |
| --- | --- | --- | --- |
|  | **QC failure** | **N.A.** |  |
| **3** | 165 |  | 165 |
| **6** | 5 |  | 5 |
| **N.A.** |  | 5 | 5 |
| **Total** | 170 | 5 | 175 |

(i) Re-analyses of the mismatches in (f), for which LANL genotypes were **5** and MuLDAS predicted **6**

| **NCBI genotype** | **REGA genotype** | | | **Subtotal** |
| --- | --- | --- | --- | --- |
|  | **QC failure** | **5** | **N.A.** |  |
| **3** | 164 |  |  | 164 |
| **5** | 2 | 2 |  | 4 |
| **6** | 5 |  |  | 5 |
| **N.A.** |  |  | 10 | 10 |
| **Total** | 171 | 2 | 10 | 183 |

(j) Re-analyses of the mismatches in (f), for which LANL genotypes were **1** and MuLDAS predicted **2**

| **NCBI genotype** | **REGA genotype** | | | **Subtotal** |
| --- | --- | --- | --- | --- |
|  | **QC failure** | **1** | **2** |  |
| **1** |  | 2 |  | 2 |
| **2** | 1 |  | 129 | 130 |
| **Total** | 1 | 2 | 129 | 132 |

**Supplementary Table 5.** Re-analysis of the benchmark test for HCV nucleotide sequences after removing batches of 3,642 sequences that had been submitted by three studies of suspicious genotype information in LANL database (see Additional File 2 Supplementary Note 3 for details).

(a) Number of gene segments per genotype before filtering

| **gene** | **1** | **2** | **3** | **4** | **5** | **6** | **Total** |
| --- | --- | --- | --- | --- | --- | --- | --- |
| **arfp** | 2,793 | 311 | 554 | 221 | 20 | 434 | 4,333 |
| **core** | 2,869 | 330 | 560 | 238 | 22 | 434 | 4,453 |
| **e1** | 11,993 | 1,016 | 1,365 | 494 | 137 | 339 | 15,344 |
| **e2** | 10,764 | 388 | 497 | 22 | 92 | 133 | 11,896 |
| **p7** | 2,067 | 8 | 16 | 20 | 1 | 44 | 2,156 |
| **ns2** | 720 | 11 | 15 | 20 | 1 | 46 | 813 |
| **ns3** | 2,763 | 65 | 251 | 22 | 107 | 46 | 3,254 |
| **ns4a** | 565 | 16 | 200 | 20 | 108 | 41 | 950 |
| **ns4b** | 700 | 11 | 41 | 20 | 108 | 41 | 921 |
| **ns5a** | 5,487 | 50 | 259 | 20 | 1 | 53 | 5,870 |
| **ns5b** | 3,260 | 453 | 504 | 442 | 122 | 334 | 5,115 |
| **okamoto** | 1,859 | 391 | 327 | 375 | 119 | 174 | 3,245 |
| **Total** | 45,840 | 3,050 | 4,589 | 1,914 | 838 | 2,119 | 58,350 |
| **Dist. Acc.** | 28,276 | 2,040 | 3,316 | 1,120 | 384 | 1,113 | 36,249 |

(b) Number of gene segments per genotype after outlierness filtering (< 2.0)

| **gene** | **1** | **2** | **3** | **4** | **5** | **6** | **Total** |
| --- | --- | --- | --- | --- | --- | --- | --- |
| **arfp** | 2,781 | 306 | 551 | 133 | 3 | 393 | 4,167 |
| **core** | 2,868 | 327 | 559 | 229 | 9 | 420 | 4,412 |
| **e1** | 11,987 | 1,010 | 1,364 | 382 | 78 | 337 | 15,158 |
| **e2** | 10,744 | 386 | 419 | 0 | 40 | 129 | 11,718 |
| **p7** | 2,067 | 8 | 10 | 0 | 1 | 34 | 2,120 |
| **ns2** | 720 | 11 | 15 | 0 | 1 | 38 | 785 |
| **ns3** | 2,761 | 65 | 250 | 0 | 4 | 45 | 3,125 |
| **ns4a** | 565 | 16 | 157 | 1 | 37 | 20 | 796 |
| **ns4b** | 697 | 11 | 35 | 0 | 3 | 41 | 787 |
| **ns5a** | 5,470 | 50 | 220 | 0 | 1 | 53 | 5,794 |
| **ns5b** | 3,212 | 383 | 465 | 4 | 3 | 317 | 4,384 |
| **okamoto** | 1,859 | 390 | 327 | 375 | 117 | 172 | 3,240 |
| **Total** | 45,731 | 2,963 | 4,372 | 1,124 | 297 | 1,999 | 56,486 |
| **Dist. Acc.** | 28,232 | 2,031 | 3,192 | 964 | 257 | 1,094 | 35,770 |

(c) Genotype prediction concordance (%) with LANL benchmark test dataset before filtering

| **gene** | **1** | **2** | **3** | **4** | **5** | **6** | **Total** |
| --- | --- | --- | --- | --- | --- | --- | --- |
| **arfp** | 99.86 | 98.07 | 99.82 | 100.00 | 100.00 | 99.54 | 99.70 |
| **core** | 99.86 | 98.18 | 99.82 | 100.00 | 100.00 | 99.77 | 99.73 |
| **e1** | 99.96 | 98.52 | 99.85 | 100.00 | 100.00 | 99.71 | 99.85 |
| **e2** | 99.75 | 100.00 | 99.40 | 100.00 | 51.09 | 100.00 | 99.37 |
| **p7** | 100.00 | 100.00 | 100.00 | 100.00 | 100.00 | 100.00 | 100.00 |
| **ns2** | 100.00 | 100.00 | 100.00 | 100.00 | 100.00 | 100.00 | 100.00 |
| **ns3** | 100.00 | 98.46 | 100.00 | 100.00 | 100.00 | 100.00 | 99.97 |
| **ns4a** | 100.00 | 100.00 | 99.00 | 90.00 | 100.00 | 100.00 | 99.58 |
| **ns4b** | 100.00 | 100.00 | 92.68 | 100.00 | 100.00 | 100.00 | 99.67 |
| **ns5a** | 99.98 | 100.00 | 100.00 | 100.00 | 100.00 | 100.00 | 99.98 |
| **ns5b** | 97.36 | 99.78 | 99.40 | 99.55 | 98.36 | 99.70 | 98.14 |
| **okamoto** | 95.32 | 100.00 | 99.08 | 99.73 | 98.32 | 100.00 | 97.13 |
| **Total** | 99.53 | 99.05 | 99.61 | 99.74 | 94.15 | 99.76 | 99.45 |

(d) Genotype prediction concordance (%) after outlierness filtering (< 2.0)

| **gene** | **1** | **2** | **3** | **4** | **5** | **6** | **Total** |
| --- | --- | --- | --- | --- | --- | --- | --- |
| **arfp** | 99.89 | 98.04 | 100.00 | 100.00 | 100.00 | 100.00 | 99.78 |
| **core** | 99.86 | 98.17 | 100.00 | 100.00 | 100.00 | 100.00 | 99.77 |
| **e1** | 99.96 | 98.51 | 99.85 | 100.00 | 100.00 | 99.70 | 99.85 |
| **e2** | 99.85 | 100.00 | 99.28 |  | 17.50 | 100.00 | 99.56 |
| **p7** | 100.00 | 100.00 | 100.00 |  | 100.00 | 100.00 | 100.00 |
| **ns2** | 100.00 | 100.00 | 100.00 |  | 100.00 | 100.00 | 100.00 |
| **ns3** | 100.00 | 98.46 | 100.00 |  | 100.00 | 100.00 | 99.97 |
| **ns4a** | 100.00 | 100.00 | 99.36 | 0.00 | 100.00 | 100.00 | 99.75 |
| **ns4b** | 100.00 | 100.00 | 91.43 |  | 100.00 | 100.00 | 99.62 |
| **ns5a** | 99.98 | 100.00 | 100.00 |  | 100.00 | 100.00 | 99.98 |
| **ns5b** | 97.48 | 99.74 | 99.57 | 75.00 | 66.67 | 99.68 | 98.02 |
| **okamoto** | 95.32 | 100.00 | 99.08 | 99.73 | 98.29 | 100.00 | 97.13 |
| **Total** | 99.57 | 99.02 | 99.68 | 99.73 | 87.88 | 99.90 | 99.50 |

(e) Confusion table (LANL on the left, MuLDAS at the top) before filtering

| **LANL** | **1** | **2** | **3** | **4** | **5** | **6** | **Total** |
| --- | --- | --- | --- | --- | --- | --- | --- |
| **1** | 45,626 | 149 | 51 | 7 | 3 | 4 | 45,840 |
| **2** | 26 | 3,021 | 0 | 2 | 0 | 1 | 3,050 |
| **3** | 9 | 6 | 4,571 | 0 | 1 | 2 | 4,589 |
| **4** | 2 | 0 | 0 | 1,909 | 1 | 2 | 1,914 |
| **5** | 19 | 7 | 7 | 0 | 789 | 16 | 838 |
| **6** | 0 | 1 | 1 | 0 | 3 | 2,114 | 2,119 |
| **Total** | 45,682 | 3,184 | 4,630 | 1,918 | 797 | 2,139 | 58,350 |

(f) Confusion table (LANL on the left, MuLDAS at the top) (outlierness < 2.0)

| **LANL** | **1** | **2** | **3** | **4** | **5** | **6** | **Total** |
| --- | --- | --- | --- | --- | --- | --- | --- |
| **1** | 45,534 | 145 | 47 | 1 | 0 | 4 | 45,731 |
| **2** | 26 | 2,934 | 0 | 2 | 0 | 1 | 2,963 |
| **3** | 9 | 3 | 4,358 | 0 | 0 | 2 | 4,372 |
| **4** | 2 | 0 | 0 | 1,121 | 0 | 1 | 1,124 |
| **5** | 14 | 7 | 4 | 0 | 261 | 11 | 297 |
| **6** | 0 | 1 | 1 | 0 | 0 | 1,997 | 1,999 |
| **Total** | 45,585 | 3,090 | 4,410 | 1,124 | 261 | 2,016 | 56,486 |

**Supplementary Table 6.** Benchmark results of HIV-1 CRF nucleotide sequences from 'nested' analysis

| **Category** | **All** | | | **Outlierness < 2.0 & *P* > 0.99** | | |
| --- | --- | --- | --- | --- | --- | --- |
|  | **Total** | **Matched** | **% acc.** | **Total** | **Matched** | **% acc.** |
| (a) by gene segment | | | | |  |  |
| **gag** | 1,458 | 961 | 65.91 | 817 | 758 | 92.78 |
| **pol** | 4,012 | 3,423 | 85.32 | 2,403 | 2,355 | 98.00 |
| **vif** | 28 | 19 | 67.86 | 20 | 14 | 70.00 |
| **vpr** | 23 | 12 | 52.17 | 7 | 7 | 100.00 |
| **tat** | 25 | 19 | 76.00 | 8 | 8 | 100.00 |
| **env** | 3,389 | 2,786 | 82.21 | 1,722 | 1,570 | 91.17 |
| **nef** | 65 | 29 | 44.62 | 17 | 16 | 94.12 |
| (b) by subtype | | | | |  |  |
| **02_AG** | 5,178 | 4,621 | 89.24 | 4,037 | 3,989 | 98.81 |
| **03_AB** | 264 | 42 | 15.91 | 94 | 0 | 0 |
| **04_cpx** | 23 | 15 | 65.22 | 4 | 4 | 100.00 |
| **05_DF** | 27 | 20 | 74.07 | 5 | 5 | 100.00 |
| **06_cpx** | 1,104 | 788 | 71.38 | 115 | 110 | 95.65 |
| **07_BC** | 586 | 467 | 79.69 | 178 | 163 | 91.57 |
| **08_BC** | 279 | 116 | 41.58 | 65 | 23 | 35.38 |
| **09_cpx** | 56 | 35 | 62.50 | 8 | 7 | 87.50 |
| **10_CD** | 161 | 62 | 38.51 | 20 | 1 | 5.00 |
| **11_cpx** | 708 | 590 | 83.33 | 275 | 273 | 99.27 |
| **12_BF** | 332 | 313 | 94.28 | 145 | 140 | 96.55 |
| **13_cpx** | 167 | 141 | 84.43 | 9 | 5 | 55.56 |
| **14_BG** | 96 | 28 | 29.17 | 31 | 3 | 9.68 |
| **15_01B** | 14 | 6 | 42.86 | 5 | 2 | 40.00 |
| **16_A2D** | 5 | 5 | 100.00 | 3 | 3 | 100.00 |
| **Total** | 9,000 | 7,249 | 80.54 | 4,994 | 4,728 | 94.67 |

**Supplementary Table 7.** Comparison with other methods for 148 cases that were discordant between MuLDAS and LANL for HIV-1 genome sequences longer than 9,000bp (Sequence set (3) in Table 5)

1. Each method’s concordance with LANL subtypes

| Sequence type | | Pure^1^ | CRF^2^ | Non-CRF^3^ | Total |
| --- | --- | --- | --- | --- | --- |
| Total number of sequences | | 7 | 24 | 117 | 148 |
| MuLDAS | Inferable^4^ | 0 | 23 | 80 | 103 |
| NCBI | Concord or inferable | 0 | 24 | 73 | 97 |
| REGA | Concord or inferable | 6 | 16 | 3 | 25 |
|  | Failed QC^5^ | 1 | 4 | 102 | 107 |
|  | No output | 0 | 4 | 1 | 5 |

^1^HIV-1 M group and CRF_01AE

^2^Other circulating recombinant forms (CRF02_AG ~ CRF16_A2D)

^3^Other recombinant forms of HIV-1

^4^If the subtype composition of gene-by-gene results of MuLDAS is congruent to the recombination pattern designated by LANL, it is counted as ‘inferable’

^5^Usually indicates that a recombination pattern is detected but with low bootscanning support

1. Pairwise concordance among four resources

| Concord or inferable | LANL | MuLDAS | NCBI |
| --- | --- | --- | --- |
| MuLDAS | 103 |  |  |
| NCBI | 97 | 102 |  |
| REGA | 25 | 26 | 17 |
