# Supplementary material for: A classification approach for genotyping viral sequences based on multidimensional scaling and linear discriminant analysis
Source: BMC Bioinformatics. 2010 Aug 21;11:434. doi: 10.1186/1471-2105-11-434 (PMC2936400; doi:10.1186/1471-2105-11-434)
Supplement: Additional file 2 — Supplementary Notes. Additional analyses and descriptions provided here include: Note 1. Comparison of 'outlierness' with the branching index in tree-based methods. Note 2. Simulation of new genotype detection by leaving out entire reference sequences of a given genotype and classifying them based on the other reference groups. Note 3. Re-analysis of discordant cases of HCV nucleotide sequences. Note 4. Test results with artificial synthetic HIV-1 nucleotide sequences. [file 1471-2105-11-434-S2.DOC]

**Supplementary Materials submitted to *BMC* *Bioinformatics***

# A classification approach for genotyping viral sequences based on multidimensional scaling and linear discriminant analysis

### Jiwoong Kim^1^, Yongju Ahn^2^, Kichan Lee, Sung Hee Park and Sangsoo Kim^§^

Department of Bioinformatics & Life Sciences, Soongsil University, Seoul, Korea 156-743

Present addresses:

^1^Equispharm Co., Ltd, Suwon, Korea 443-766. ^2^Macrogen Inc., Seoul, Korea 153-023

^§^Corresponding author

### Additional file 2 – Supplementary Notes

Additional analyses and descriptions provided here include:

Note 1. Comparison of ‘outlierness’ with the branching index in tree-based methods

Note 2. Simulation of new genotype detection by leaving out entire reference sequences of a given genotype and classifying them based on the other reference groups.

Note 3. Re-analysis of discordant cases of HCV nucleotide sequences

Note 4. Test results with artificial synthetic HIV-1 nucleotide sequences

### Supplementary Note 1. Comparison of ‘outlierness’ with the branching index in tree-based methods

.

The branching index (BI) [1, 2] is for tree-based methods as 'outlierness' is for MuLDAS, a geometric method. BI and 'outlierness' have a similar purpose of signalling potential problematic sequences. However, the measuring schemes are different. BI measures the relative distance from the node of the query to the most recent common ancestor (MRCA) of the subtype cluster. The distance is divided by the total branch length of the subtype cluster. Since the cutoff is based on this relative distance, it may concern the proximity of the query to the subtype of interest compared to the next nearest subtype. Similar cutoffs can be used for viruses of different degree of divergence within a subtype, as long as the relative location of the branching node of the query does not change too much. On the other hand, 'outlierness' in MuLDAS concerns the relative divergence of a subtype. It does not care how close other subtypes approach to the query, as that is accounted by the posterior probabilities of the subtypes. We have compared these two different approaches using a sequence (AY036374) that has been used as an example of hypermutation in the last paragraph of Results & Discussion. MuLDAS reported this sequence having 'outlierness' greater than 10, indicating extremely divergent. In the Neighbor-Joining tree produced by REGA, it was located as the outermost branch. Consequently, we cannot calculate BI in this case. We also tried PhyloPlace from LANL that serves a web-based BI method. The server did not report any result. According to the server manager, it is not designed to be used with hypermutated sequences. We also checked the MuLDAS results of the six HIV-1 recombinant sequences that were used in the original development of BI [1]. As these sequences have been deposited with GenBank, we were able to retrieve their pre-calculated results from our database server. Please see the following table retrieved from our database server for these sequences:

| **Retrieval from the Database of Pre-calcuated Genotyping/Subtyping Results** |
| --- |

| Virus: HIV-1 / Sequence Type: Nucleotide  Multiple Substitution Correction Algorithm: Jukes-Cantor  All sites that include gaps were deleted.  **Gene by Gene Subtype Prediction Summary (**[**Download as an EXCEL file**](http://www2.muldas.org/MuLDAS/record.cgi?TARGET=hiv1_nucleotide&q=AF075701AF075702AY352654AY352655AY352656AY352657&RESTRICTION=&DOWNLOAD=)**)**   | NCBI GI | ACCESSION | Gene | Start | Stop | Length | LANL Subtype | Major Subtype | Nested Analysis | Final Decision | | --- | --- | --- | --- | --- | --- | --- | --- | --- | --- | | [5668880](http://www2.muldas.org/MuLDAS/record.cgi?TARGET=hiv1_nucleotide&q=5668880) | AF075701 | [gag](http://www2.muldas.org/MuLDAS/record.cgi?TARGET=hiv1_nucleotide&q=AF075701#gag.major) | 110 | 1616 | 963 | [A1D](http://www.hiv.lanl.gov/components/sequence/HIV/combined_search_s_tree/d_search.comp?sa_genbankaccession=AF075701) | D | D | D (Unambiguous Nested) | | [pol](http://www2.muldas.org/MuLDAS/record.cgi?TARGET=hiv1_nucleotide&q=AF075701#pol.major) | 1411 | 4420 | 2703 | D | D | D (Unambiguous Nested) | | [vif](http://www2.muldas.org/MuLDAS/record.cgi?TARGET=hiv1_nucleotide&q=AF075701#vif.major) | 4364 | 4943 | 522 | D | D | D (Unambiguous Nested) | | [vpr](http://www2.muldas.org/MuLDAS/record.cgi?TARGET=hiv1_nucleotide&q=AF075701#vpr.major) | 4882 | 5173 | 147 | D | D | D (Major agrees Nested with high confidence) | | [5tat](http://www2.muldas.org/MuLDAS/record.cgi?TARGET=hiv1_nucleotide&q=AF075701#5tat.major) | 5153 | 5364 | 185 | D | D | D (Unambiguous Nested) | | [vpu](http://www2.muldas.org/MuLDAS/record.cgi?TARGET=hiv1_nucleotide&q=AF075701#vpu.major) | 5396 | 5629 | 95 | D | D | D (Unambiguous Nested) | | [env](http://www2.muldas.org/MuLDAS/record.cgi?TARGET=hiv1_nucleotide&q=AF075701#env.major) | 5546 | 8069 | 1588 | A | A | A (Unambiguous Nested) | | [nef](http://www2.muldas.org/MuLDAS/record.cgi?TARGET=hiv1_nucleotide&q=AF075701#nef.major) | 8070 | 8691 | 246 | D | D | D (Unambiguous Nested) | | [ltr](http://www2.muldas.org/MuLDAS/record.cgi?TARGET=hiv1_nucleotide&q=AF075701#ltr.major) | 8362 | 8879 | 256 | D | D | D (Unambiguous Nested) | | [5305477](http://www2.muldas.org/MuLDAS/record.cgi?TARGET=hiv1_nucleotide&q=5305477) | AF075702 | [gag](http://www2.muldas.org/MuLDAS/record.cgi?TARGET=hiv1_nucleotide&q=AF075702#gag.major) | 111 | 1611 | 963 | [A1CD](http://www.hiv.lanl.gov/components/sequence/HIV/combined_search_s_tree/d_search.comp?sa_genbankaccession=AF075702) | A | A | A (Major agrees Nested with high confidence) | | [pol](http://www2.muldas.org/MuLDAS/record.cgi?TARGET=hiv1_nucleotide&q=AF075702#pol.major) | 1406 | 4415 | 2703 | A | A | A (Unambiguous Nested) | | [vif](http://www2.muldas.org/MuLDAS/record.cgi?TARGET=hiv1_nucleotide&q=AF075702#vif.major) | 4359 | 4938 | 522 | A | A | A (Unambiguous Nested) | | [vpr](http://www2.muldas.org/MuLDAS/record.cgi?TARGET=hiv1_nucleotide&q=AF075702#vpr.major) | 4877 | 5171 | 147 | A | A | A (Unambiguous Nested) | | [5tat](http://www2.muldas.org/MuLDAS/record.cgi?TARGET=hiv1_nucleotide&q=AF075702#5tat.major) | 5151 | 5362 | 185 | A | A | A (Unambiguous Nested) | | [vpu](http://www2.muldas.org/MuLDAS/record.cgi?TARGET=hiv1_nucleotide&q=AF075702#vpu.major) | 5405 | 5638 | 95 | A | A | A (Major agrees Nested with high confidence) | | [env](http://www2.muldas.org/MuLDAS/record.cgi?TARGET=hiv1_nucleotide&q=AF075702#env.major) | 5555 | 8117 | 1588 | A | A | A (Unambiguous Nested) | | [nef](http://www2.muldas.org/MuLDAS/record.cgi?TARGET=hiv1_nucleotide&q=AF075702#nef.major) | 8118 | 8742 | 246 | C | C | C (Unambiguous Nested) | | [ltr](http://www2.muldas.org/MuLDAS/record.cgi?TARGET=hiv1_nucleotide&q=AF075702#ltr.major) | 8410 | 8955 | 256 | C | C | C (Unambiguous Nested) | | [38679131](http://www2.muldas.org/MuLDAS/record.cgi?TARGET=hiv1_nucleotide&q=38679131) | AY352654 | [gag](http://www2.muldas.org/MuLDAS/record.cgi?TARGET=hiv1_nucleotide&q=AY352654#gag.major) | 154 | 1684 | 963 | [GKU](http://www.hiv.lanl.gov/components/sequence/HIV/combined_search_s_tree/d_search.comp?sa_genbankaccession=AY352654) | A | 04_cpx | A (Highly confident Major) | | [pol](http://www2.muldas.org/MuLDAS/record.cgi?TARGET=hiv1_nucleotide&q=AY352654#pol.major) | 1458 | 4488 | 2703 | J | 06_cpx | Undecided | | [vif](http://www2.muldas.org/MuLDAS/record.cgi?TARGET=hiv1_nucleotide&q=AY352654#vif.major) | 4432 | 5011 | 522 | J | 13_cpx | Undecided | | [vpr](http://www2.muldas.org/MuLDAS/record.cgi?TARGET=hiv1_nucleotide&q=AY352654#vpr.major) | 4950 | 5241 | 147 | A | A | A (Unambiguous Nested) | | [5tat](http://www2.muldas.org/MuLDAS/record.cgi?TARGET=hiv1_nucleotide&q=AY352654#5tat.major) | 5221 | 5432 | 185 | K | K | Undecided | | [vpu](http://www2.muldas.org/MuLDAS/record.cgi?TARGET=hiv1_nucleotide&q=AY352654#vpu.major) | 5475 | 5708 | 95 | B | 08_BC | Undecided | | [env](http://www2.muldas.org/MuLDAS/record.cgi?TARGET=hiv1_nucleotide&q=AY352654#env.major) | 5625 | 8220 | 1588 | H | H | Undecided | | [nef](http://www2.muldas.org/MuLDAS/record.cgi?TARGET=hiv1_nucleotide&q=AY352654#nef.major) | 8221 | 8860 | 246 | G | G | G (Major agrees Nested with high confidence) | | [ltr](http://www2.muldas.org/MuLDAS/record.cgi?TARGET=hiv1_nucleotide&q=AY352654#ltr.major) | 8528 | 9074 | 256 | K | K | Undecided | | [38679140](http://www2.muldas.org/MuLDAS/record.cgi?TARGET=hiv1_nucleotide&q=38679140) | AY352655 | [gag](http://www2.muldas.org/MuLDAS/record.cgi?TARGET=hiv1_nucleotide&q=AY352655#gag.major) | 108 | 1617 | 963 | [GKU](http://www.hiv.lanl.gov/components/sequence/HIV/combined_search_s_tree/d_search.comp?sa_genbankaccession=AY352655) | A | 04_cpx | A (Highly confident Major) | | [pol](http://www2.muldas.org/MuLDAS/record.cgi?TARGET=hiv1_nucleotide&q=AY352655#pol.major) | 1412 | 4421 | 2703 | J | 06_cpx | Undecided | | [vif](http://www2.muldas.org/MuLDAS/record.cgi?TARGET=hiv1_nucleotide&q=AY352655#vif.major) | 4365 | 4944 | 522 | J | 13_cpx | Undecided | | [vpr](http://www2.muldas.org/MuLDAS/record.cgi?TARGET=hiv1_nucleotide&q=AY352655#vpr.major) | 4883 | 5174 | 147 | A | A | Undecided | | [5tat](http://www2.muldas.org/MuLDAS/record.cgi?TARGET=hiv1_nucleotide&q=AY352655#5tat.major) | 5154 | 5365 | 185 | J | 11_cpx | 11_cpx (Unambiguous Nested) | | [vpu](http://www2.muldas.org/MuLDAS/record.cgi?TARGET=hiv1_nucleotide&q=AY352655#vpu.major) | 5407 | 5640 | 95 | F | 08_BC | Undecided | | [env](http://www2.muldas.org/MuLDAS/record.cgi?TARGET=hiv1_nucleotide&q=AY352655#env.major) | 5557 | 8215 | 1580 | H | 04_cpx | Undecided | | [nef](http://www2.muldas.org/MuLDAS/record.cgi?TARGET=hiv1_nucleotide&q=AY352655#nef.major) | 8216 | 8851 | 246 | G | K | Undecided | | [ltr](http://www2.muldas.org/MuLDAS/record.cgi?TARGET=hiv1_nucleotide&q=AY352655#ltr.major) | 8519 | 9066 | 256 | K | K | Undecided | | [38679148](http://www2.muldas.org/MuLDAS/record.cgi?TARGET=hiv1_nucleotide&q=38679148) | AY352656 | [gag](http://www2.muldas.org/MuLDAS/record.cgi?TARGET=hiv1_nucleotide&q=AY352656#gag.major) | 153 | 1650 | 963 | [A1D](http://www.hiv.lanl.gov/components/sequence/HIV/combined_search_s_tree/d_search.comp?sa_genbankaccession=AY352656) | A | A | A (Unambiguous Nested) | | [pol](http://www2.muldas.org/MuLDAS/record.cgi?TARGET=hiv1_nucleotide&q=AY352656#pol.major) | 1442 | 4454 | 2703 | A | A | A (Unambiguous Nested) | | [vif](http://www2.muldas.org/MuLDAS/record.cgi?TARGET=hiv1_nucleotide&q=AY352656#vif.major) | 4398 | 4977 | 522 | A | A | A (Unambiguous Nested) | | [vpr](http://www2.muldas.org/MuLDAS/record.cgi?TARGET=hiv1_nucleotide&q=AY352656#vpr.major) | 4916 | 5207 | 147 | D | D | D (Unambiguous Nested) | | [5tat](http://www2.muldas.org/MuLDAS/record.cgi?TARGET=hiv1_nucleotide&q=AY352656#5tat.major) | 5187 | 5398 | 185 | A | A | A (Unambiguous Nested) | | [vpu](http://www2.muldas.org/MuLDAS/record.cgi?TARGET=hiv1_nucleotide&q=AY352656#vpu.major) | 5435 | 5668 | 95 | A | A | Undecided | | [env](http://www2.muldas.org/MuLDAS/record.cgi?TARGET=hiv1_nucleotide&q=AY352656#env.major) | 5585 | 8147 | 1583 | D | D | Undecided | | [nef](http://www2.muldas.org/MuLDAS/record.cgi?TARGET=hiv1_nucleotide&q=AY352656#nef.major) | 8148 | 8763 | 246 | A | A | A (Unambiguous Nested) | | [ltr](http://www2.muldas.org/MuLDAS/record.cgi?TARGET=hiv1_nucleotide&q=AY352656#ltr.major) | 8431 | 8977 | 256 | A | A | A (Unambiguous Nested) | | [38679157](http://www2.muldas.org/MuLDAS/record.cgi?TARGET=hiv1_nucleotide&q=38679157) | AY352657 | [gag](http://www2.muldas.org/MuLDAS/record.cgi?TARGET=hiv1_nucleotide&q=AY352657#gag.major) | 153 | 1647 | 963 | [A1D](http://www.hiv.lanl.gov/components/sequence/HIV/combined_search_s_tree/d_search.comp?sa_genbankaccession=AY352657) | A | A | A (Unambiguous Nested) | | [pol](http://www2.muldas.org/MuLDAS/record.cgi?TARGET=hiv1_nucleotide&q=AY352657#pol.major) | 1442 | 4451 | 2703 | A | A | A (Unambiguous Nested) | | [vif](http://www2.muldas.org/MuLDAS/record.cgi?TARGET=hiv1_nucleotide&q=AY352657#vif.major) | 4395 | 4974 | 522 | D | 05_DF | Undecided | | [vpr](http://www2.muldas.org/MuLDAS/record.cgi?TARGET=hiv1_nucleotide&q=AY352657#vpr.major) | 4913 | 5204 | 147 | D | D | D (Unambiguous Nested) | | [5tat](http://www2.muldas.org/MuLDAS/record.cgi?TARGET=hiv1_nucleotide&q=AY352657#5tat.major) | 5184 | 5395 | 185 | A | 01_AE | Undecided | | [vpu](http://www2.muldas.org/MuLDAS/record.cgi?TARGET=hiv1_nucleotide&q=AY352657#vpu.major) | 5438 | 5671 | 95 | 01_AE | 01_AE | 01_AE (Unambiguous Nested) | | [env](http://www2.muldas.org/MuLDAS/record.cgi?TARGET=hiv1_nucleotide&q=AY352657#env.major) | 5588 | 8144 | 1588 | D | D | D (Unambiguous Nested) | | [nef](http://www2.muldas.org/MuLDAS/record.cgi?TARGET=hiv1_nucleotide&q=AY352657#nef.major) | 8145 | 8769 | 246 | D | D | Undecided | | [ltr](http://www2.muldas.org/MuLDAS/record.cgi?TARGET=hiv1_nucleotide&q=AY352657#ltr.major) | 8437 | 8984 | 256 | A | A | A (Major agrees Nested with high confidence) | |
| --- | --- | --- | --- | --- | --- | --- | --- | --- | --- | --- | --- | --- | --- | --- | --- | --- | --- | --- | --- | --- | --- | --- | --- | --- | --- | --- | --- | --- | --- | --- | --- | --- | --- | --- | --- | --- | --- | --- | --- | --- | --- | --- | --- | --- | --- | --- | --- | --- | --- | --- | --- | --- | --- | --- | --- | --- | --- | --- | --- | --- | --- | --- | --- | --- | --- | --- | --- | --- | --- | --- | --- | --- | --- | --- | --- | --- | --- | --- | --- | --- | --- | --- | --- | --- | --- | --- | --- | --- | --- | --- | --- | --- | --- | --- | --- | --- | --- | --- | --- | --- | --- | --- | --- | --- | --- | --- | --- | --- | --- | --- | --- | --- | --- | --- | --- | --- | --- | --- | --- | --- | --- | --- | --- | --- | --- | --- | --- | --- | --- | --- | --- | --- | --- | --- | --- | --- | --- | --- | --- | --- | --- | --- | --- | --- | --- | --- | --- | --- | --- | --- | --- | --- | --- | --- | --- | --- | --- | --- | --- | --- | --- | --- | --- | --- | --- | --- | --- | --- | --- | --- | --- | --- | --- | --- | --- | --- | --- | --- | --- | --- | --- | --- | --- | --- | --- | --- | --- | --- | --- | --- | --- | --- | --- | --- | --- | --- | --- | --- | --- | --- | --- | --- | --- | --- | --- | --- | --- | --- | --- | --- | --- | --- | --- | --- | --- | --- | --- | --- | --- | --- | --- | --- | --- | --- | --- | --- | --- | --- | --- | --- | --- | --- | --- | --- | --- | --- | --- | --- | --- | --- | --- | --- | --- | --- | --- | --- | --- | --- | --- | --- | --- | --- | --- | --- | --- | --- | --- | --- | --- | --- | --- | --- | --- | --- | --- | --- | --- | --- | --- | --- | --- | --- | --- | --- | --- | --- | --- | --- | --- | --- | --- | --- | --- | --- | --- | --- | --- | --- | --- | --- | --- | --- | --- | --- | --- | --- | --- | --- | --- | --- | --- | --- | --- | --- | --- | --- | --- | --- | --- | --- | --- | --- | --- | --- | --- | --- | --- | --- | --- | --- | --- | --- | --- | --- | --- | --- | --- | --- | --- | --- | --- | --- | --- | --- | --- | --- | --- | --- | --- | --- | --- | --- | --- | --- | --- | --- | --- | --- | --- | --- | --- | --- | --- | --- | --- | --- | --- | --- | --- | --- | --- | --- | --- | --- | --- | --- | --- | --- | --- | --- | --- | --- | --- | --- | --- | --- | --- | --- | --- | --- | --- | --- | --- | --- | --- | --- | --- | --- | --- | --- | --- | --- | --- | --- | --- | --- | --- | --- | --- | --- | --- | --- | --- | --- | --- | --- |

Four of them had a non-complex recombination pattern that involved two to three subtypes. The recombinant segments that occupied the major portion of a gene segment were confidently subtyped by MuLDAS, while some short segments were not detected as their contribution to the distance may have not been large enough to inflate 'outlierness'. The BI values for these four sequences were also above their threshold for confident classification. The other two sequences showed extremely complex recombination patterns and their BI values far below the threshold. MuLDAS also reported that most of the gene segments of these sequences cannot be subtyped with confidence and left them as 'Undecided'.

### Supplementary Note 2. Simulation of new genotype detection by leaving out entire reference sequences of a given genotype and classifying them based on the other reference groups

.

MuLDAS is not a class discovery method but a method classifying a query sequence into one of the classes. However, it is based on a phylogenetic principle of clustering based on genetic distance between sequences. If a host of sequences emerged as a truly new genotype, they should form a nice cluster, distinct from the rest of the groups. If MuLDAS is applied to such cases, they may be nominally classified to one of the groups with very high posterior probabilities. However they do not mix with any other group but form a cluster themselves. Consequently their ‘outlierness’ value from the nominal group would be very high.

This situation is simulated by leaving out entire reference sequences of a given genotype and classifying them based on the other reference groups only. For a given gene segment, a pairwise distance matrix was calculated between all the reference sequences. Multidimensional scaling mapped the sequences into a multidimensional space. A linear discriminant model was built based the coordinates of the sequences from all but one genotype. The genotypes of the sequences left out were then predicted based the model. The maximum a posterior probability and the outlierness value from the nominal group were evaluated for each sequence. The entire process was repeated for each genotype. The representative distributions of *O* values are shown below as boxplots. As many of the *O* values were extremely large (out of scale), we plotted 1/*O* instead. In HIV-1, most subtypes had very large *O* values, except for CRF01_AE, which is rather closer to the subtype A.

Also in HCV, none of the genotypes had *O* values less than 1.0, indicating distinct clustering. Depending on gene segments, some genotypes had *O* values between 1.0 and 2.0.

###

### Supplementary Note 3. Re-analysis of discordant cases of HCV nucleotide sequences

A close examination of the benchmark test result of HCV nucleotide sequences with LANL genotype information showed several points noteworthy (Additional File 1 Supplementary Table 4). Firstly, *e1* and *e2* sequences belonging to genotype 4 or 5 showed concordance rate lower than 60%, even though there were at least several hundreds of sequences in each category. Secondly, there were particular types of discordances that were frequent: for example, 1=>2, 4=>1, 5=>3, or 5=>6, where LANL=>MuLDAS. It is likely that these two kinds of patterns are actually from the same cases. For the latter kind of discordance, we re-analyzed their genotypes using NCBI Genotyping Tool and REGA. Since NCBI Genotyping Tool does not summarize its sliding-window result, we picked the best scoring one. Many sequences did not pass the quality control step built in REGA. Typically they are due to recombination or outlier sequences. The re-analysis results are presented in Additional File 1 Supplementary Table 4(g-j). Whenever the majority of sequences passed the REGA QC, the concordance between NCBI Genotyping Tool and REGA were excellent and furthermore their results favoured those of MuLDAS rather than those of LANL (g and j). In the other two cases (h and i), most of the sequences did not pass REGA QC, but NCBI tool was successfully genotyped them. In one the cases, the NCBI results were in agreement with MuLDAS (h). In the remaining case, the NCBI results were discordant to either LANL or MuLDAS (i). However, even in this case, the composition of the NCBI sliding-window result showed substantial portion of the sequence had the genotype as predicted by MuLDAS. In summary, among the four routes of information, LANL was the outlier.

We realized that the majority of these sequences have been submitted by the authors of three different publications, as shown below:

| PubMed ID | Total number of sequences submitted in GenBank | Total number of sequences in our re-analysis |
| --- | --- | --- |
| 16707577 | 2672 | 786 |
| 16781757 | 1337 | 90 |
| 17314160 | 563 | 25 |

As the table shows, the bulk of these sequences may be genotyped well. If we remove only the discordant ones from these sources and re-calculate the benchmark statistics, it would not be balanced. Instead we removed all the sequences from these publications and re-calculated the statistics (Supplementary Table 5 in Additional File 1).

### Supplementary Note 4. Test results with artificial synthetic HIV-1 nucleotide sequences

.

As a classification tool, MuLDAS has been developed to assign a subtype among a set of known subtypes, and thus not designed to detect a new subtype or recombination pattern. However, MuLDAS may hint some important clues for the analysis of these outlier sequences in terms of outlierness value and a set of posterior probabilities as well as the complex subtype pattern along the sequence. Here we provide the summary of the test runs of MuLDAS with artificial HIV-1 sequences interwoven with two subtypes. The authors of jpHMM [3, 4] provide some test sets used to detect recombinants available from <http://jphmm.gobics.de/>. Each of the test sequences was a synthetic one interwoven with chunks of another subtype. Three different sizes of the inserted chunks were used to create the test sequences. Our approach that split the input by gene boundary missed most of the small recombinant chunks (300bp). For the larger chunks (500 or 1000 bp), MuLDAS were able to identify the inserted subtypes. Since MuLDAS is not designed to detect *de novo* recombination, but to classify the query to the most similar CRF. If either the recombination spot or subtype composition of the query is substantially different from the common CRFs, it would not perform well. This implies that we may need a sliding-window capability in order to detect such recombinations with MuLDAS. We plan to implement it in the near future. Please see the following table that summarizes the test runs. Please refer to the web site mentioned above for details of the test sequences.

| **description** | **gag** | **pol** | **vif** | **vpr** | **5tat** | **5rev** | **vpu** | **env** | **3tat** | **3rev** | **nef** | **ltr** | **summary** |
| --- | --- | --- | --- | --- | --- | --- | --- | --- | --- | --- | --- | --- | --- |
| (a) At every 1000 bp, chunks of 1000 bp are substituted by the second subtype |  |  |  |  |  |  |  |  |  |  |  |  |  |
| A1B_1000_A1.KZ.2002.02KZYUZ300425.EF589041_B.CA.1996.WC10P_9.AY314051 796 9464 | B | A | B | B | B | C | B | A | C | A | B | B |  |
| BA1_1000_B.CA.1996.WC10C_10.AY314061_A1.KE.1986.ML013_10.AY322184 555 9639 | A | B | A | A | A | B | B | B | B | B | A | A |  |
| A1C_1000_A1.KE.2001.ML1901.EU110087_C.ZA.2000.C_ZA_1069MB.AY585268 673 9635 | C | A | C | C | C | A | A | A | C | A | C | C |  |
| CA1_1000_C.BW.1996.96BW0407.AF110963_A1.AU.2004.PS1044_Day177.DQ676873 512 9414 | A | C | A | A | C | C | C | C | C | C | A | A |  |
| A1D_1000_A1.RW.1993.93RW037A.AB287378_D.CD.1983.NDK.A34828 1 9636 | A | D | A | D | D | D | A | A | A | A | A | D |  |
| DA1_1000_D.TD.1999.MN011.AJ519488_A1.IT.2002.60000.EU861977 783 9719 | A | J | G | A | G | B | D | D | D | D | A | A |  |
| A1F1_1000_A1.KZ.2002.02KZYUZ300413.EF589040_F1.-.-.MVP_30846.EU446022 796 9635 | F | K | F | F | F | C | A | A | A | A | F | F |  |
| F1A1_1000_F1.-.-.MVP_30846.EU446022_A1.BY.1997.97BL006.AF193275 650 9632 | A | F | A | A | 01_AE | A | F | F | F | F | A | A |  |
| A1G_1000_A1.UG.-.UG031.AB098330_G.KE.1993.HH8793_1_1.AF061640 1 9640 | A | G | J | G | G | G | A | A | A | A | A | G |  |
| GA1_1000_G.GH.2003.GHNJ175.AB231893_A1.UG.1992.92UG037.U51190 1 9590 | G | A | J | A | A | A | G | G | G | G | 01_AE | A |  |
| A101_AE_1000_A1.KZ.2002.02KZKAR300435.EF589042_01_AE.JP.1993.NH25.AB070352 796 9719 | 01_AE | 01_AE | 01_AE | 01_AE | 01_AE | 01_AE | A | A | A | A | 01_AE | 01_AE |  |
| 01_AEA1_1000_01_AE.JP.-.DR5032.AB253723_A1.KZ.2002.02KZPAV300497.EF589039 1 9603 | 01_AE | A | 01_AE | A | A | 01_AE | A | 01_AE | 01_AE | 01_AE | 01_AE | A |  |
| BC_1000_B.US.1989.5096_89.AY835750_C.IN.-.VB39.EF694032 1 9629 | B | C | B | C | C | C | B | B | B | B | B | C |  |
| CB_1000_C.BW.1996.96BW15C05.AF110975_B.AU.2002.PS3002_Day0.DQ676883 512 9411 | B | C | B | B | B | C | C | C | C | C | B | B |  |
| BF1_1000_B.FR.-.AX032749_F1.-.-.MVP_30846.EU446022 1 9635 | B | F | B | F | F | F | B | B | B | B | B | F |  |
| F1B_1000_F1.-.-.MVP_30846.EU446022_B.AU.2003.PS3002_Day199.DQ676884 650 9411 | B | F | B | B | B | A | F | K | F | F | B | B |  |
| BG_1000_B.US.1982.5048_82.AY835759_G.GH.2003.GHNJ175.AB231893 1 9632 | B | G | B | G | G | G | B | B | B | B | B | K |  |
| GB_1000_G.ES.2005.ES.EU786670_B.US.-.AC_59_41_Days_Seq1_fa.DQ127548 561 9425 | B | G | B | B | B | A | G | G | G | G | B | B |  |
| B01_AE_1000_B.US.-.AC160_T2_Day_83_Dom.EU616640_01_AE.JP.-.DR1236.AB253695 790 9632 | 01_AE | B | 01_AE | 01_AE | 01_AE | B | B | B | B | B | 01_AE | 01_AE |  |
| 01_AEB_1000_01_AE.JP.-.DR1873.AB253647_B.US.1985.WCIPR.U69585 1 9636 | 01_AE | B | 01_AE | B | B | B | A | 01_AE | 01_AE | 01_AE | 01_AE | B |  |
| CD_1000_C.ZM.2002.02ZM112.AB254144_D.CD.1983.ELI.A14116 1 9637 | C | D | C | D | D | A | C | C | C | C | C | D |  |
| DC_1000_D.UG.1998.98UG57130.AF484504_C.ZM.2002.02ZM112.AB254143 796 9632 | C | D | C | C | C | D | D | D | D | D | C | C |  |
| CF1_1000_C.BW.1996.96BW01.AF110960_F1.-.-.MVP_30846.EU446022 512 9635 | F | C | F | F | F | C | C | C | C | C | C | F |  |
| F1C_1000_F1.-.-.MVP_30846.EU446022_C.ZA.-.99ZALT21.EU293446 650 9719 | C | K | C | C | K | A | F | K | F | F | C | C |  |
| CG_1000_C.BW.1996.96BW06J7.AF290029_G.GH.2003.GHNJ175.AB231893 1 9632 | C | G | J | G | G | G | C | C | J | C | C | G |  |
| GC_1000_G.GH.2003.03GH175G.AB287003_C.ZM.2002.02ZMDB.AB254152 1 9632 | G | C | J | C | C | C | A | G | J | G | 01_AE | C |  |
| C01_AE_1000_C.ZA.1998.TV001.BD437615_01_AE.JP.-.DR0492.AB253688 1 9632 | C | 01_AE | C | 01_AE | 01_AE | 01_AE | C | C | C | C | C | 01_AE |  |
| 01_AEC_1000_01_AE.JP.-.DR1236.AB253702_C.ZA.-.99ZALT42.EU293448 1 9719 | 01_AE | C | 01_AE | C | C | A | 01_AE | 01_AE | 01_AE | 01_AE | 01_AE | J |  |
| DF1_1000_D.TD.1999.MN012.AJ519489_F1.-.-.MVP_30846.EU446022 783 9635 | F | K | F | F | F | B | D | D | D | D | F | F |  |
| F1D_1000_F1.-.-.MVP_30846.EU446022_D.CD.1983.ELI.A14116 650 9637 | D | K | D | D | D | A | F | K | F | F | D | D |  |
| DG_1000_D.CD.1983.NDK.A34828_G.GH.2003.GHNJ175.AB231893 455 9632 | G | J | G | G | G | D | D | D | D | D | G | G |  |
| GD_1000_G.KE.1993.HH8793_1_1.AF061640_D.CD.1983.ELI.A14116 618 9637 | D | J | D | D | D | A | G | G | G | G | D | D |  |
| D01_AE_1000_D.CD.1983.ELI.A07108_01_AE.JP.-.DR2192.AB253707 455 9632 | 01_AE | J | 01_AE | 01_AE | 01_AE | D | D | D | D | D | 01_AE | 01_AE |  |
| 01_AED_1000_01_AE.JP.-.DR5032.AB253718_D.CD.1983.ELI.A14116 1 9637 | 01_AE | D | 01_AE | D | D | A | A | 01_AE | 01_AE | 01_AE | 01_AE | D |  |
| F1G_1000_F1.-.-.MVP_30846.EU446022_G.GH.2003.GHNJ175.AB231893 650 9632 | G | F | G | G | G | 01_AE | F | K | F | F | G | G |  |
| GF1_1000_G.ES.2005.ES.EU786670_F1.-.-.MVP_30846.EU446022 561 9635 | F | G | F | F | F | A | G | G | G | G | F | F |  |
| F101_AE_1000_F1.-.-.MVP_30846.EU446022_01_AE.JP.-.DR1741.AB253642 650 9632 | 01_AE | K | 01_AE | 01_AE | 01_AE | 01_AE | F | F | F | F | 01_AE | 01_AE |  |
| 01_AEF1_1000_01_AE.JP.-.DR2192.AB253706_F1.-.-.MVP_30846.EU446022 1 9635 | 01_AE | F | 01_AE | F | F | F | 01_AE | 01_AE | 01_AE | 01_AE | 01_AE | A |  |
| G01_AE_1000_G.GH.2003.GHNJ175.AB231893_01_AE.JP.-.DR1741.AB253645 1 9632 | G | 01_AE | J | 01_AE | 01_AE | 01_AE | G | G | G | G | 01_AE | 01_AE |  |
| 01_AEG_1000_01_AE.TH.1990.CM235.AF259954_G.GH.2003.03GH175G.AB287003 1 9632 | 01_AE | G | 01_AE | G | G | G | 01_AE | 01_AE | 01_AE | 01_AE | 01_AE | G |  |
| (b) At every 1500 bp, chunks of 300 bp are substituted by the second subtype |  |  |  |  |  |  |  |  |  |  |  |  |  |
| A1B_1500_300_A1.KZ.2002.02KZYUZ300425.EF589041_B.CA.1996.WC10P_9.AY314051 796 9603 | A | A | B | A | A | C | B | A | C | A | F | A |  |
| BA1_1500_300_B.CA.1996.WC10C_10.AY314061_A1.KE.1986.ML013_10.AY322184 555 9464 | B | B | A | B | B | B | B | B | B | B | B | B |  |
| A1C_1500_300_A1.KE.2001.ML1901.EU110087_C.ZA.2000.C_ZA_1069MB.AY585268 673 9591 | A | A | C | A | A | A | A | A | C | A | A | A |  |
| CA1_1500_300_C.BW.1996.96BW0407.AF110963_A1.AU.2004.PS1044_Day177.DQ676873 512 9529 | C | C | A | C | C | C | C | C | C | C | C | C |  |
| A1D_1500_300_A1.RW.1993.93RW037A.AB287378_D.CD.1983.NDK.A34828 1 9632 | A | A | A | J | A | A | A | A | A | A | A | A | fail |
| DA1_1500_300_D.TD.1999.MN011.AJ519488_A1.IT.2002.60000.EU861977 783 9618 | D | D | G | D | D | B | D | D | D | D | D | D | fail |
| A1F1_1500_300_A1.KZ.2002.02KZYUZ300413.EF589040_F1.-.-.MVP_30846.EU446022 796 9603 | A | A | K | A | A | C | A | A | A | A | A | A | fail |
| F1A1_1500_300_F1.-.-.MVP_30846.EU446022_A1.BY.1997.97BL006.AF193275 650 9635 | F | F | A | F | F | A | F | F | F | F | A | F |  |
| A1G_1500_300_A1.UG.-.UG031.AB098330_G.KE.1993.HH8793_1_1.AF061640 1 9632 | A | A | A | A | A | A | A | A | A | A | A | A |  |
| GA1_1500_300_G.GH.2003.GHNJ175.AB231893_A1.UG.1992.92UG037.U51190 1 9632 | G | G | G | A | G | G | G | G | G | G | 01_AE | G |  |
| A101_AE_1500_300_A1.KZ.2002.02KZKAR300435.EF589042_01_AE.JP.1993.NH25.AB070352 796 9603 | A | A | 01_AE | A | 01_AE | C | A | A | A | A | A | A |  |
| 01_AEA1_1500_300_01_AE.JP.-.DR5032.AB253723_A1.KZ.2002.02KZPAV300497.EF589039 1 9632 | 01_AE | 01_AE | 01_AE | G | 01_AE | 01_AE | 01_AE | 01_AE | 01_AE | 01_AE | 01_AE | 01_AE | fail |
| BC_1500_300_B.US.1989.5096_89.AY835750_C.IN.-.VB39.EF694032 1 9719 | B | B | C | B | B | B | B | B | B | B | B | B |  |
| CB_1500_300_C.BW.1996.96BW15C05.AF110975_B.AU.2002.PS3002_Day0.DQ676883 512 9529 | C | C | B | C | C | C | C | C | C | C | C | C |  |
| BF1_1500_300_B.FR.-.AX032749_F1.-.-.MVP_30846.EU446022 1 9719 | B | B | K | B | B | B | B | B | B | B | B | B | fail |
| F1B_1500_300_F1.-.-.MVP_30846.EU446022_B.AU.2003.PS3002_Day199.DQ676884 650 9635 | F | F | K | F | F | A | F | F | F | F | F | F | fail |
| BG_1500_300_B.US.1982.5048_82.AY835759_G.GH.2003.GHNJ175.AB231893 1 9719 | B | B | B | G | B | B | B | B | B | B | B | B |  |
| GB_1500_300_G.ES.2005.ES.EU786670_B.US.-.AC_59_41_Days_Seq1_fa.DQ127548 561 9509 | G | G | B | G | G | G | G | G | G | G | K | G |  |
| B01_AE_1500_300_B.US.-.AC160_T2_Day_83_Dom.EU616640_01_AE.JP.-.DR1236.AB253695 790 9417 | B | B | 01_AE | B | B | B | B | B | B | B | B | B |  |
| 01_AEB_1500_300_01_AE.JP.-.DR1873.AB253647_B.US.1985.WCIPR.U69585 1 9632 | 01_AE | 01_AE | 01_AE | B | 01_AE | 01_AE | 01_AE | 01_AE | 01_AE | 01_AE | 01_AE | 01_AE |  |
| CD_1500_300_C.ZM.2002.02ZM112.AB254144_D.CD.1983.ELI.A14116 1 9632 | C | C | C | J | C | C | C | C | C | C | C | C | fail |
| DC_1500_300_D.UG.1998.98UG57130.AF484504_C.ZM.2002.02ZM112.AB254143 796 9603 | D | D | C | D | D | D | D | D | D | D | D | D |  |
| CF1_1500_300_C.BW.1996.96BW01.AF110960_F1.-.-.MVP_30846.EU446022 512 9529 | C | C | H | C | C | C | C | C | C | C | C | C | fail |
| F1C_1500_300_F1.-.-.MVP_30846.EU446022_C.ZA.-.99ZALT21.EU293446 650 9635 | F | F | C | F | F | A | F | F | F | F | F | F |  |
| CG_1500_300_C.BW.1996.96BW06J7.AF290029_G.GH.2003.GHNJ175.AB231893 1 9719 | C | C | G | C | C | C | C | C | C | C | C | C |  |
| GC_1500_300_G.GH.2003.03GH175G.AB287003_C.ZM.2002.02ZMDB.AB254152 1 9632 | G | G | G | C | G | G | G | G | G | G | 01_AE | G |  |
| C01_AE_1500_300_C.ZA.1998.TV001.BD437615_01_AE.JP.-.DR0492.AB253688 1 9719 | C | C | C | 01_AE | C | C | C | C | C | C | C | C |  |
| 01_AEC_1500_300_01_AE.JP.-.DR1236.AB253702_C.ZA.-.99ZALT42.EU293448 1 9632 | 01_AE | 01_AE | 01_AE | C | 01_AE | 01_AE | 01_AE | 01_AE | 01_AE | 01_AE | 01_AE | 01_AE |  |
| DF1_1500_300_D.TD.1999.MN012.AJ519489_F1.-.-.MVP_30846.EU446022 783 9618 | D | D | K | D | D | B | D | D | D | D | D | D | fail |
| F1D_1500_300_F1.-.-.MVP_30846.EU446022_D.CD.1983.ELI.A14116 650 9635 | F | F | D | F | F | A | F | F | F | F | F | F |  |
| DG_1500_300_D.CD.1983.NDK.A34828_G.GH.2003.GHNJ175.AB231893 455 9636 | D | D | G | D | D | D | D | D | D | D | D | D |  |
| GD_1500_300_G.KE.1993.HH8793_1_1.AF061640_D.CD.1983.ELI.A14116 618 9640 | G | G | D | G | G | G | G | G | G | G | G | G |  |
| D01_AE_1500_300_D.CD.1983.ELI.A07108_01_AE.JP.-.DR2192.AB253707 455 9637 | D | D | 01_AE | D | D | D | D | D | D | D | D | D |  |
| 01_AED_1500_300_01_AE.JP.-.DR5032.AB253718_D.CD.1983.ELI.A14116 1 9632 | 01_AE | 01_AE | 01_AE | D | 01_AE | 01_AE | 01_AE | 01_AE | 01_AE | 01_AE | 01_AE | 01_AE |  |
| F1G_1500_300_F1.-.-.MVP_30846.EU446022_G.GH.2003.GHNJ175.AB231893 650 9635 | F | F | G | F | F | A | F | F | F | F | A | F |  |
| GF1_1500_300_G.ES.2005.ES.EU786670_F1.-.-.MVP_30846.EU446022 561 9509 | G | G | K | G | G | G | G | G | G | G | G | G | fail |
| F101_AE_1500_300_F1.-.-.MVP_30846.EU446022_01_AE.JP.-.DR1741.AB253642 650 9635 | F | F | 01_AE | F | F | A | F | F | F | F | A | F |  |
| 01_AEF1_1500_300_01_AE.JP.-.DR2192.AB253706_F1.-.-.MVP_30846.EU446022 1 9632 | 01_AE | 01_AE | 01_AE | K | 01_AE | 01_AE | 01_AE | 01_AE | 01_AE | 01_AE | 01_AE | 01_AE | fail |
| G01_AE_1500_300_G.GH.2003.GHNJ175.AB231893_01_AE.JP.-.DR1741.AB253645 1 9632 | G | G | G | 01_AE | G | G | G | G | G | G | 01_AE | G |  |
| 01_AEG_1500_300_01_AE.TH.1990.CM235.AF259954_G.GH.2003.03GH175G.AB287003 1 9719 | 01_AE | 01_AE | 01_AE | G | 01_AE | 01_AE | 01_AE | 01_AE | 01_AE | 01_AE | 01_AE | 01_AE |  |
| (c) At every 1500 bp, chunks of 500 bp are substituted by the second subtype |  |  |  |  |  |  |  |  |  |  |  |  |  |
| A1B_1500_500_A1.KZ.2002.02KZYUZ300425.EF589041_B.CA.1996.WC10P_9.AY314051 796 9500 | A | A | A | B | B | C | B | A | C | A | A | A |  |
| BA1_1500_500_B.CA.1996.WC10C_10.AY314061_A1.KE.1986.ML013_10.AY322184 555 9464 | J | B | B | A | A | B | B | B | B | B | B | B |  |
| A1C_1500_500_A1.KE.2001.ML1901.EU110087_C.ZA.2000.C_ZA_1069MB.AY585268 673 9635 | A | A | A | C | C | A | A | A | C | A | A | A |  |
| CA1_1500_500_C.BW.1996.96BW0407.AF110963_A1.AU.2004.PS1044_Day177.DQ676873 512 9500 | C | C | C | A | C | C | C | C | C | C | C | C |  |
| A1D_1500_500_A1.RW.1993.93RW037A.AB287378_D.CD.1983.NDK.A34828 1 9636 | A | A | A | A | D | D | A | A | A | A | A | A |  |
| DA1_1500_500_D.TD.1999.MN011.AJ519488_A1.IT.2002.60000.EU861977 783 9719 | D | D | D | A | G | B | D | D | D | D | D | D |  |
| A1F1_1500_500_A1.KZ.2002.02KZYUZ300413.EF589040_F1.-.-.MVP_30846.EU446022 796 9635 | A | A | A | F | F | C | A | A | A | A | A | A |  |
| F1A1_1500_500_F1.-.-.MVP_30846.EU446022_A1.BY.1997.97BL006.AF193275 650 9632 | F | F | F | A | 01_AE | A | F | F | F | F | F | F |  |
| A1G_1500_500_A1.UG.-.UG031.AB098330_G.KE.1993.HH8793_1_1.AF061640 1 9640 | A | A | A | A | G | G | A | A | A | A | A | A |  |
| GA1_1500_500_G.GH.2003.GHNJ175.AB231893_A1.UG.1992.92UG037.U51190 1 9590 | G | G | G | G | A | A | G | G | G | G | 01_AE | G |  |
| A101_AE_1500_500_A1.KZ.2002.02KZKAR300435.EF589042_01_AE.JP.1993.NH25.AB070352 796 9719 | 01_AE | A | G | 01_AE | 01_AE | 01_AE | A | A | A | A | A | A |  |
| 01_AEA1_1500_500_01_AE.JP.-.DR5032.AB253723_A1.KZ.2002.02KZPAV300497.EF589039 1 9603 | 01_AE | 01_AE | 01_AE | 01_AE | A | 01_AE | A | 01_AE | 01_AE | 01_AE | 01_AE | 01_AE |  |
| BC_1500_500_B.US.1989.5096_89.AY835750_C.IN.-.VB39.EF694032 1 9629 | B | J | B | B | C | C | B | B | B | B | B | B |  |
| CB_1500_500_C.BW.1996.96BW15C05.AF110975_B.AU.2002.PS3002_Day0.DQ676883 512 9500 | C | C | C | B | B | C | C | C | C | C | C | C |  |
| BF1_1500_500_B.FR.-.AX032749_F1.-.-.MVP_30846.EU446022 1 9635 | B | B | B | B | F | F | B | B | B | B | B | B |  |
| F1B_1500_500_F1.-.-.MVP_30846.EU446022_B.AU.2003.PS3002_Day199.DQ676884 650 9500 | F | F | F | B | B | A | F | F | F | F | F | F |  |
| BG_1500_500_B.US.1982.5048_82.AY835759_G.GH.2003.GHNJ175.AB231893 1 9632 | B | B | B | B | G | G | B | B | B | B | B | B |  |
| GB_1500_500_G.ES.2005.ES.EU786670_B.US.-.AC_59_41_Days_Seq1_fa.DQ127548 561 9500 | G | G | G | B | B | A | G | G | G | G | G | G |  |
| B01_AE_1500_500_B.US.-.AC160_T2_Day_83_Dom.EU616640_01_AE.JP.-.DR1236.AB253695 790 9417 | B | B | B | 01_AE | 01_AE | B | B | B | B | B | B | B |  |
| 01_AEB_1500_500_01_AE.JP.-.DR1873.AB253647_B.US.1985.WCIPR.U69585 1 9636 | 01_AE | 01_AE | 01_AE | 01_AE | B | B | A | 01_AE | 01_AE | 01_AE | 01_AE | 01_AE |  |
| CD_1500_500_C.ZM.2002.02ZM112.AB254144_D.CD.1983.ELI.A14116 1 9637 | C | C | C | C | D | A | C | C | C | C | C | C |  |
| DC_1500_500_D.UG.1998.98UG57130.AF484504_C.ZM.2002.02ZM112.AB254143 796 9632 | J | D | D | C | C | D | D | D | D | D | D | D |  |
| CF1_1500_500_C.BW.1996.96BW01.AF110960_F1.-.-.MVP_30846.EU446022 512 9635 | C | C | C | F | F | C | C | C | C | C | C | C |  |
| F1C_1500_500_F1.-.-.MVP_30846.EU446022_C.ZA.-.99ZALT21.EU293446 650 9719 | F | F | F | C | K | A | F | F | F | F | F | F |  |
| CG_1500_500_C.BW.1996.96BW06J7.AF290029_G.GH.2003.GHNJ175.AB231893 1 9632 | C | C | C | C | G | G | C | C | C | C | C | C |  |
| GC_1500_500_G.GH.2003.03GH175G.AB287003_C.ZM.2002.02ZMDB.AB254152 1 9632 | G | G | G | G | C | C | A | G | G | G | 01_AE | G |  |
| C01_AE_1500_500_C.ZA.1998.TV001.BD437615_01_AE.JP.-.DR0492.AB253688 1 9632 | C | C | C | C | 01_AE | 01_AE | C | C | C | C | C | C |  |
| 01_AEC_1500_500_01_AE.JP.-.DR1236.AB253702_C.ZA.-.99ZALT42.EU293448 1 9719 | 01_AE | 01_AE | 01_AE | 01_AE | C | A | 01_AE | 01_AE | 01_AE | 01_AE | 01_AE | 01_AE |  |
| DF1_1500_500_D.TD.1999.MN012.AJ519489_F1.-.-.MVP_30846.EU446022 783 9635 | K | D | D | F | F | B | D | D | D | D | D | D |  |
| F1D_1500_500_F1.-.-.MVP_30846.EU446022_D.CD.1983.ELI.A14116 650 9637 | F | F | F | D | D | A | F | F | F | F | F | F |  |
| DG_1500_500_D.CD.1983.NDK.A34828_G.GH.2003.GHNJ175.AB231893 455 9632 | J | D | D | G | G | D | D | D | D | D | D | D |  |
| GD_1500_500_G.KE.1993.HH8793_1_1.AF061640_D.CD.1983.ELI.A14116 618 9637 | G | G | G | D | D | A | G | G | G | G | G | G |  |
| D01_AE_1500_500_D.CD.1983.ELI.A07108_01_AE.JP.-.DR2192.AB253707 455 9632 | D | D | D | 01_AE | 01_AE | D | D | D | D | D | D | D |  |
| 01_AED_1500_500_01_AE.JP.-.DR5032.AB253718_D.CD.1983.ELI.A14116 1 9637 | 01_AE | 01_AE | 01_AE | 01_AE | D | A | A | 01_AE | 01_AE | 01_AE | 01_AE | 01_AE |  |
| F1G_1500_500_F1.-.-.MVP_30846.EU446022_G.GH.2003.GHNJ175.AB231893 650 9632 | F | F | F | G | G | 01_AE | F | F | F | F | F | F |  |
| GF1_1500_500_G.ES.2005.ES.EU786670_F1.-.-.MVP_30846.EU446022 561 9635 | G | G | G | F | F | A | G | G | G | G | G | G |  |
| F101_AE_1500_500_F1.-.-.MVP_30846.EU446022_01_AE.JP.-.DR1741.AB253642 650 9632 | F | F | F | 01_AE | 01_AE | 01_AE | F | F | F | F | F | F |  |
| 01_AEF1_1500_500_01_AE.JP.-.DR2192.AB253706_F1.-.-.MVP_30846.EU446022 1 9635 | 01_AE | 01_AE | 01_AE | 01_AE | F | F | 01_AE | 01_AE | 01_AE | 01_AE | 01_AE | 01_AE |  |
| G01_AE_1500_500_G.GH.2003.GHNJ175.AB231893_01_AE.JP.-.DR1741.AB253645 1 9632 | G | G | G | G | 01_AE | 01_AE | G | G | G | G | 01_AE | G |  |
| 01_AEG_1500_500_01_AE.TH.1990.CM235.AF259954_G.GH.2003.03GH175G.AB287003 1 9632 | 01_AE | 01_AE | 01_AE | 01_AE | G | G | 01_AE | 01_AE | 01_AE | 01_AE | 01_AE | 01_AE |  |

### References

1. Wilbe K, Saminen M, Laukkanen T, McCutchan F, Ray SC, Albert J, Leitner T: **Characterization of novel recombinant HIV-1 genomes using the branching index**. *Virology* 2003, **316**:116-25.
2. Hraber P, Kuiken C, Waugh M, Geer S, Bruno WJ, Leitner T: **Classification of hepatitis C virus and human immunodeficiency virus-1 sequences with the branching index**. *J Gen Virol* 2008, **89**:2098-107.
3. Schultz AK, Zhang M, Leitner T, Kuiken C, Korber B, Morgenstern B, Stanke M: **A jumping profile Hidden Markov Model and applications to recombination sites in HIV and HCV genomes**. *BMC Bioinformatics* 2006, **7**:265.
4. Zhang M, Schultz AK, Calef C, Kuiken C, Leitner T, Korber B, Morgenstern B, Stanke M: **jpHMM at GOBICS: a web server to detect genomic recombinations in HIV-1**. *Nucleic Acids Research* 2006, **34**:W463-5.
