# Supplementary material for: A classification approach for genotyping viral sequences based on multidimensional scaling and linear discriminant analysis
Source: BMC Bioinformatics. 2010 Aug 21;11:434. doi: 10.1186/1471-2105-11-434 (PMC2936400; doi:10.1186/1471-2105-11-434)
Supplement: Additional file 3 — Supplementary Figures. Figure 1. Screenshots of MuLDAS web server for subtyping HIV-1 sequences. Figure 2. LOOCV error rates in sliding windows for HIV-1 nucleotide sequences. Figure 3. LOOCV error rates in sliding windows for HCV nucleotide sequences. Figure 4. The scatter plots of LOOCV error rate by sequence length for (a) HIV-1 and (b) HCV nucleotide sequences. Figure 5. The density distributions of the outlierness value, O, and the corresponding false discovery rates from the benchmark results for HIV-1 and HCV nucleotide sequences after removing the HCV sequences of suspicious genotypes. Figure 6. The plots of false discovery rates at each step of the proposed process for HIV-1 subtype decision. [file 1471-2105-11-434-S3.DOC]

**Supplementary Materials submitted to *BMC* *Bioinformatics***

A classification approach for genotyping viral sequences based on multidimensional scaling and linear discriminant analysis

Jiwoong Kim^1^, Yongju Ahn^2^, Kichan Lee, Sung Hee Park and Sangsoo Kim^*^

Department of Bioinformatics & Life Sciences, Soongsil University, Seoul, Korea 156-743.

Present addresses: ^1^Equispharm Co., Ltd, Suwon, Korea 443-766. ^2^Macrogen Inc., Seoul, Korea 153-023

*Contact sskimb@ssu.ac.kr

# Supplementary Figures

## Figure 1 - Screenshots of MuLDAS web server for subtyping HIV-1 sequences

1. Input; (b)-(d) the first through the last pages of the output.

## Figure 2 – LOOCV error rates in sliding windows for HIV-1 nucleotide sequences

1. *gag*, (b) *pol*, (c) *vif*, (d) *tat*, (e) *env*, and (f) *nef*

## Figure 3 – LOOCV error rates in sliding windows for HCV nucleotide sequences

1. *arfp*, (b) *core*, (c) *e1*, (d) *e2*, (e) *p7* (f) *ns2*, (g) *ns3*, (h) *ns4a*, (i) *ns4b*, (j) *ns5a*, (k) *ns5b*, (l) *okamoto*,

## Figure 4 - The scatter plots of LOOCV error rate by sequence length for (a) HIV-1 and (b) HCV nucleotide sequences

The sequence datasets used in this plots comprised of 169,015 and 79,936 gene segments longer than 50bp for HIV-1 and HCV, respectively. The horizontal axis represents the size of the query sequence, while the vertical axis represents the corresponding LOOCV error rate (%). Since the plot involved too many points to be displayed by regular scatter plot routines, we used a high volume scatter plot routine, hexbin, available from the statistical package R. This routine bins the bivariate data into 30x30 hexagonal bins and the number of data points is colored as shown on the right panel.

## Figure 5 - The density distributions of the outlierness value, *O*, and the corresponding false discovery rates from the benchmark results for HIV-1 and HCV nucleotide sequences after removing the HCV sequences of suspicious genotypes

For HIV-1 all the sequences used in the benchmark test were used (Table 1). For HCV the sequences from the submissions containing suspicious genotype information were removed (see Supplementary Table 5 in Additional File 1 and Supplementary Note 3 in Additional File 2). The *O* values were surveyed and plotted as the histograms that were separately normalized for the cases concordant with (bar) and discordant to (line) LANL genotypes/subtypes. After filtering out the cases having *O* > cutoff, the discordant ones were counted as false positives. The false positive rates and the proportion of the sequences retained (coverage) were plotted against the *O* cutoff for HIV-1 (c) and HCV (d) sequences. The suggested cutoff is shown by a dashed line.

## Figure 6 - The plots of false discovery rates at each step of the proposed process for HIV-1 subtype decision

For all 162,669 HIV-1 nucleotide gene segments (including recombinant forms), the subtype predictions discordant to LANL were treated as false positives at a given *O* value cutoff. The false discovery rate (FDR) and the proportion that were retained (coverage) were overlaid for the following steps: (a) for the cases where P > 0.99 with *O_nested_* cutoff; (b) for *O_nested_* > 2.0, subtype(major) = subtype(nested), and P < 0.99 with *O_major_* cutoff; (c) all the other but P < 0.99 with *O_major_* cutoff.


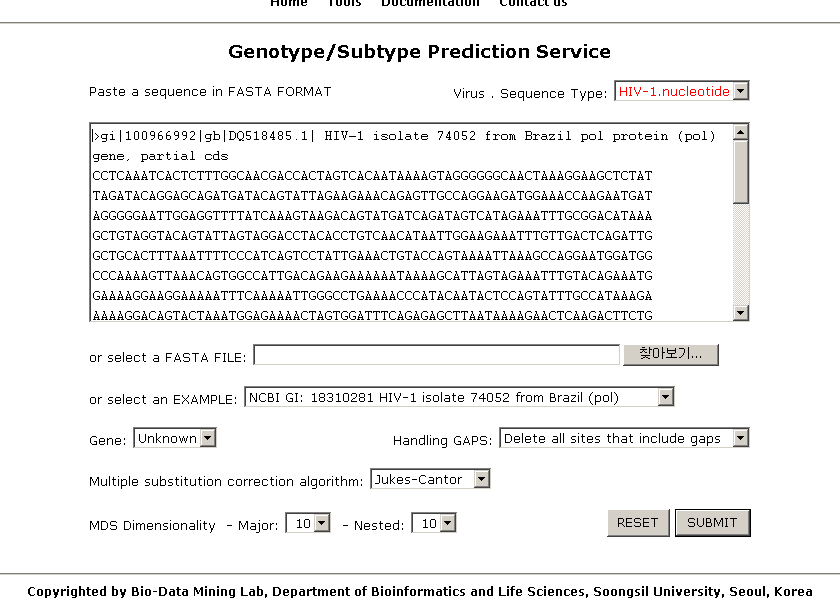


Figure 1(a)


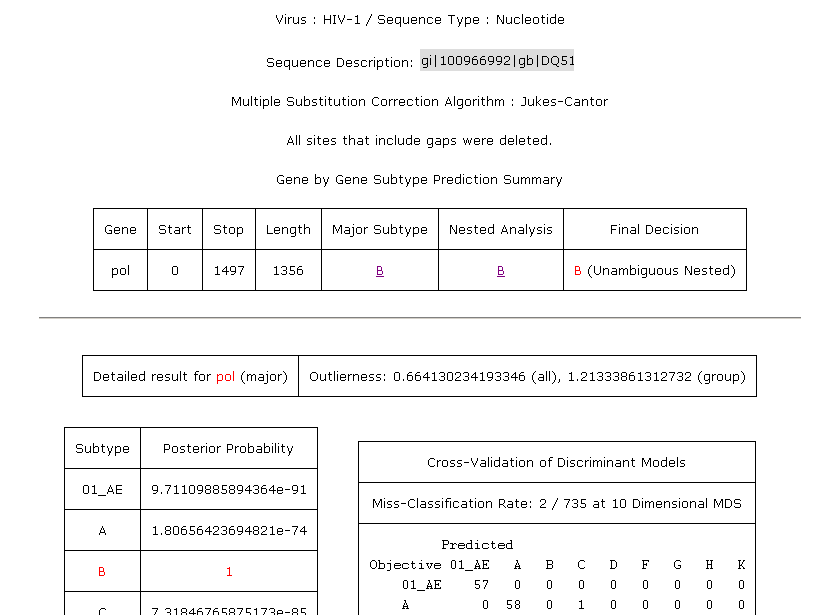


Figure 1(b)


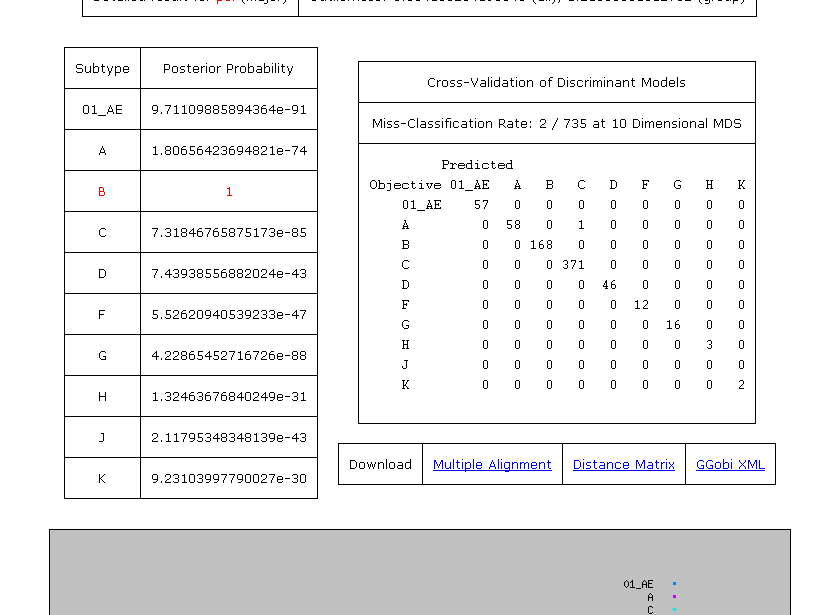


Figure 1(c)


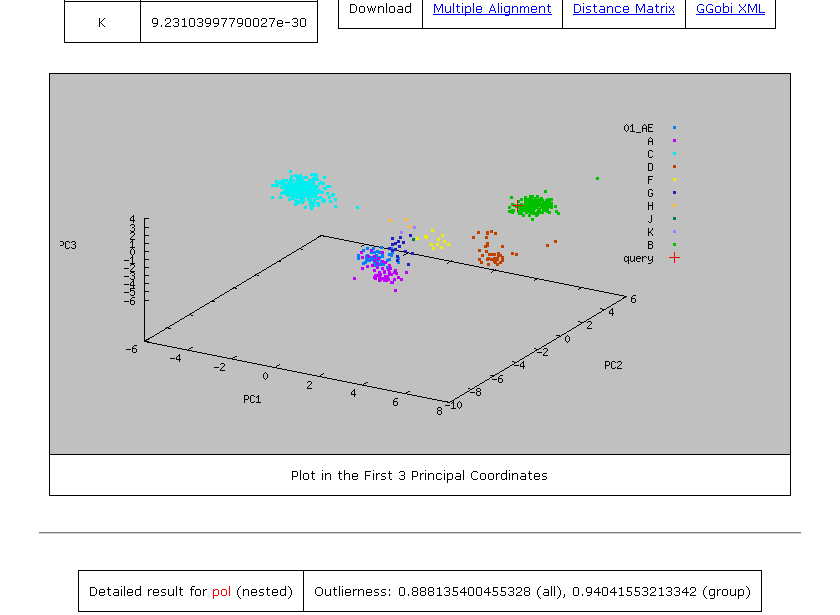


Figure 1(d)


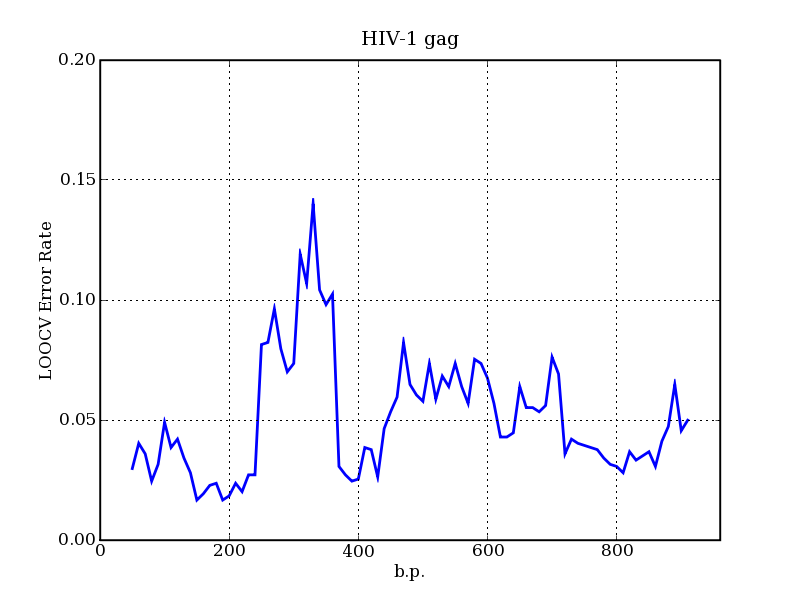


Figure 2(a)


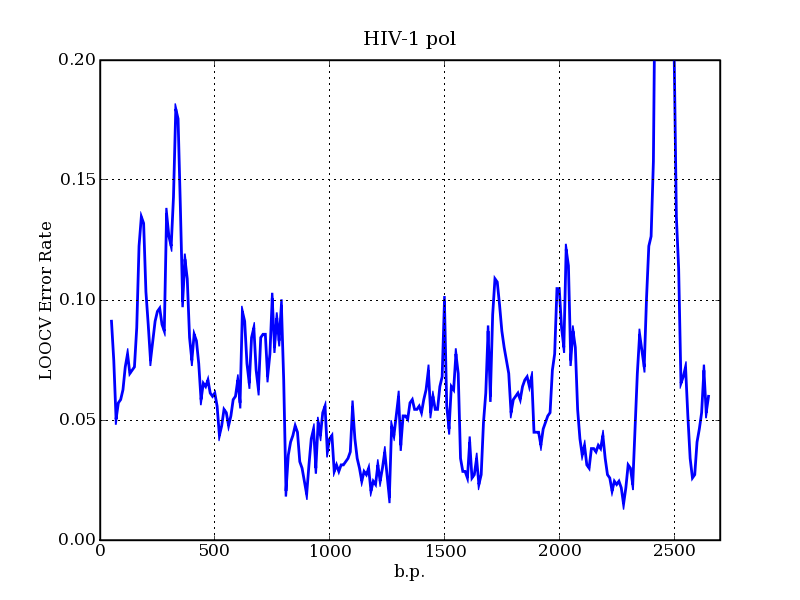


Figure 2(b)


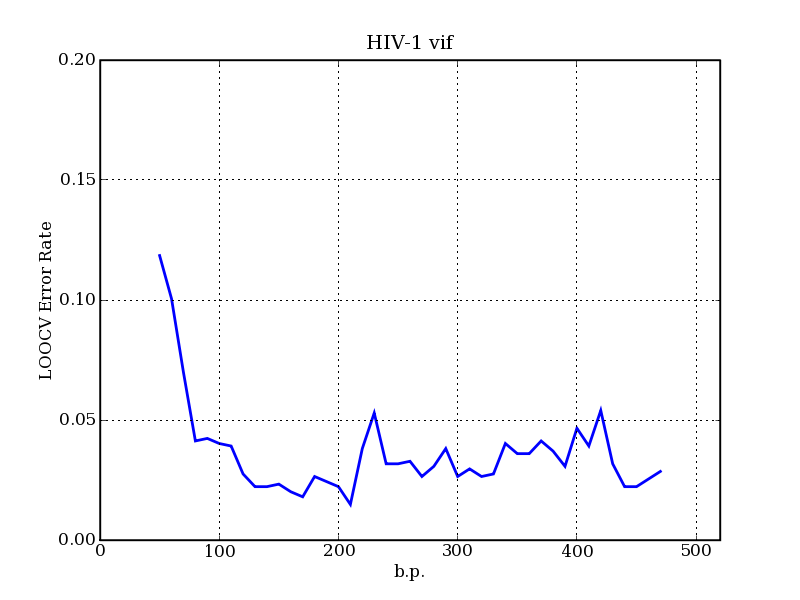


Figure 2(c)


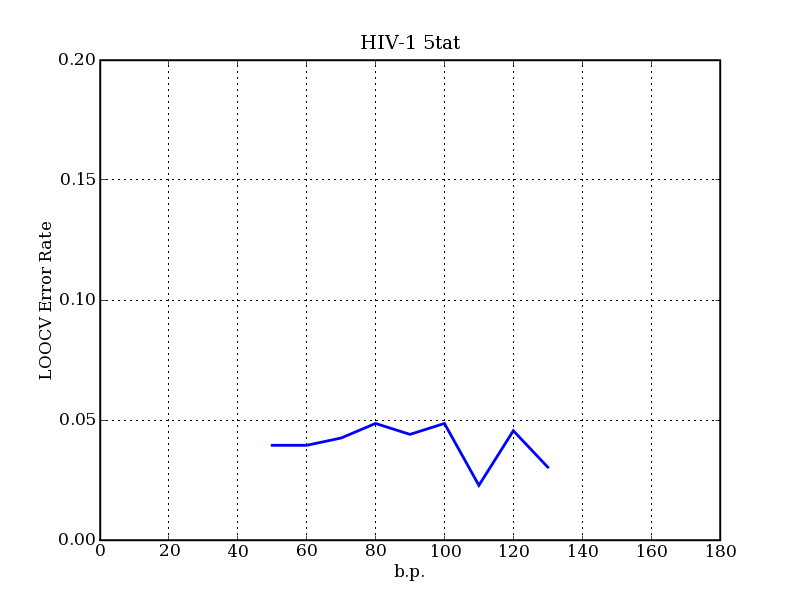


Figure 2(d)


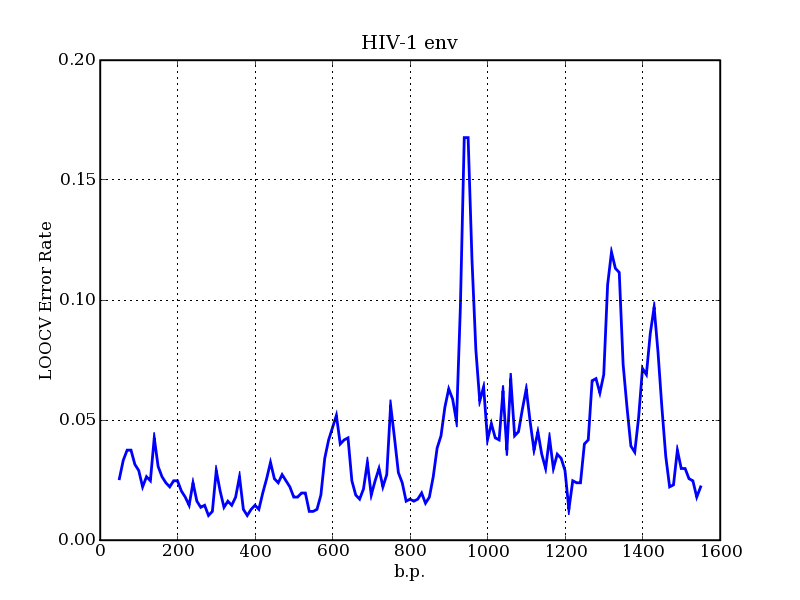


Figure 2(e)


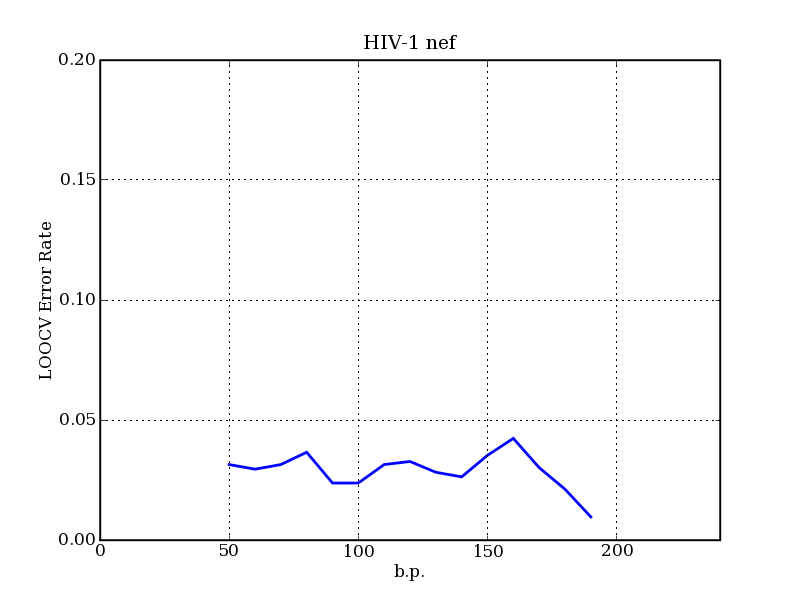


Figure 2(f)


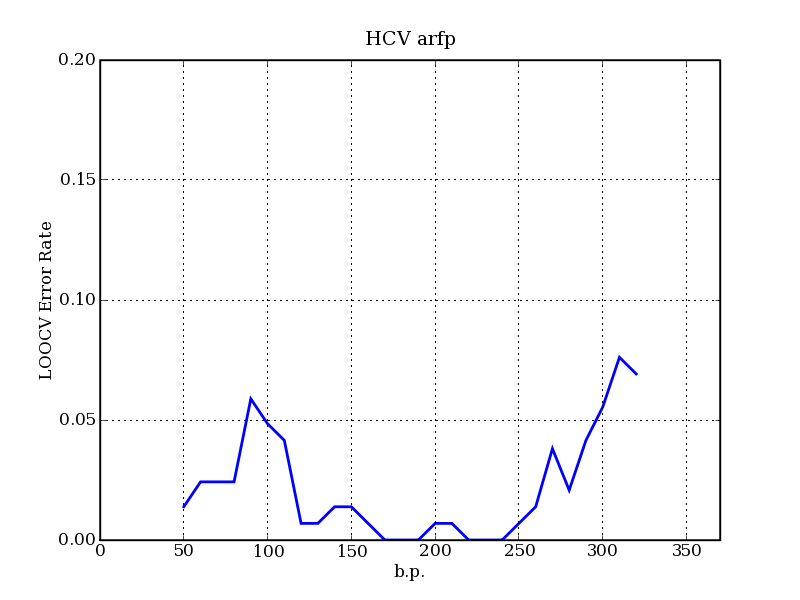


Figure 3(a)


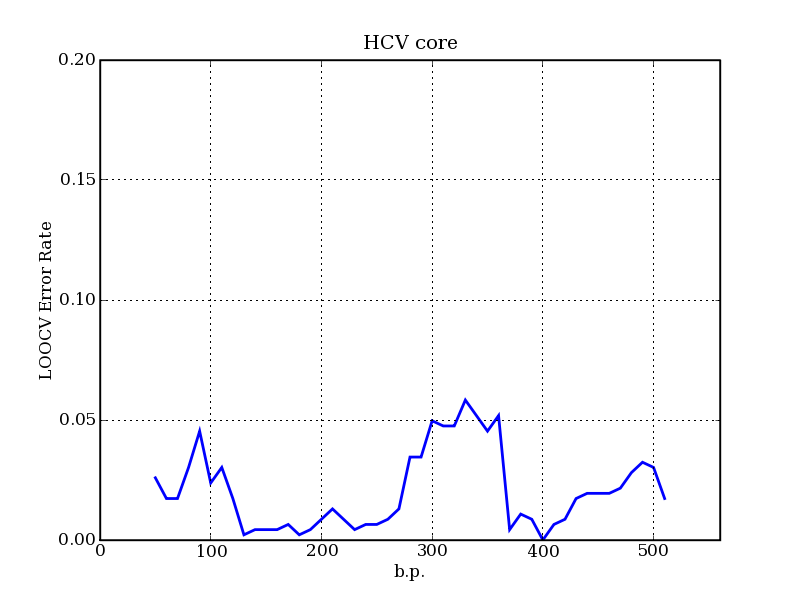


Figure 3(b)


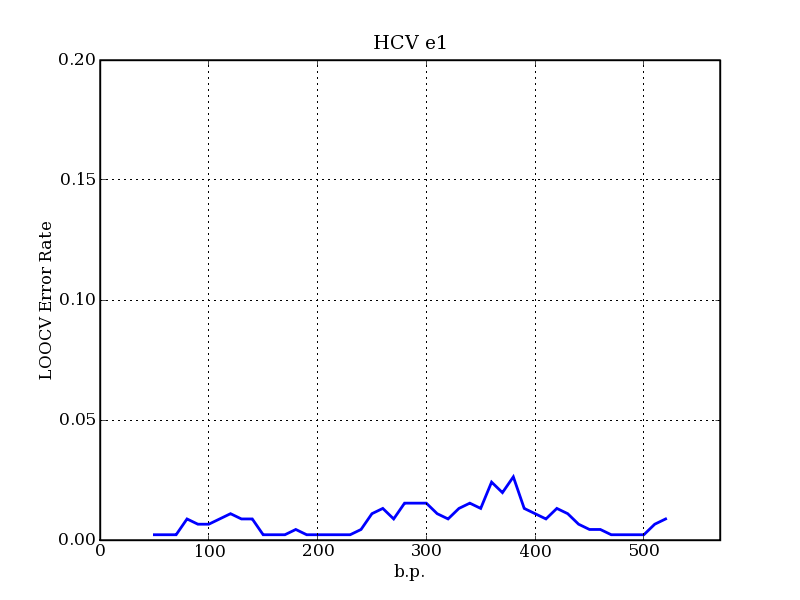


Figure 3(c)


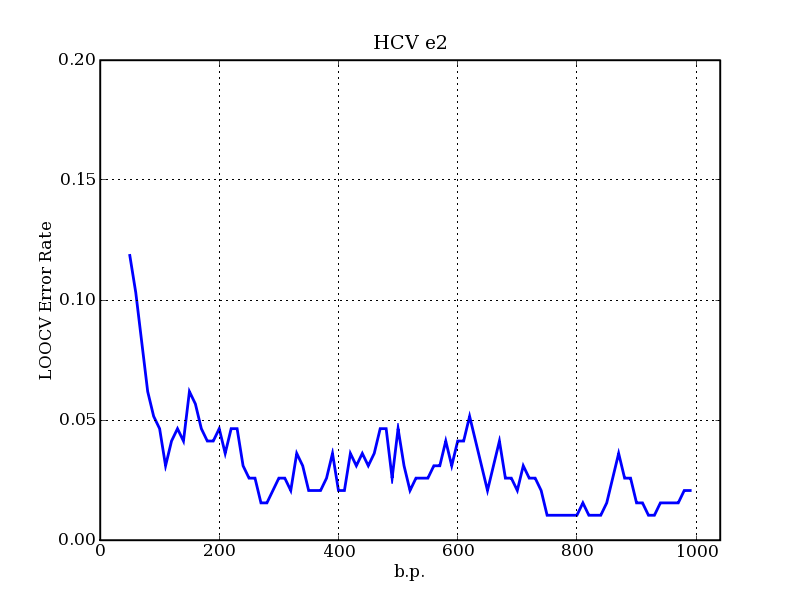


Figure 3(d)


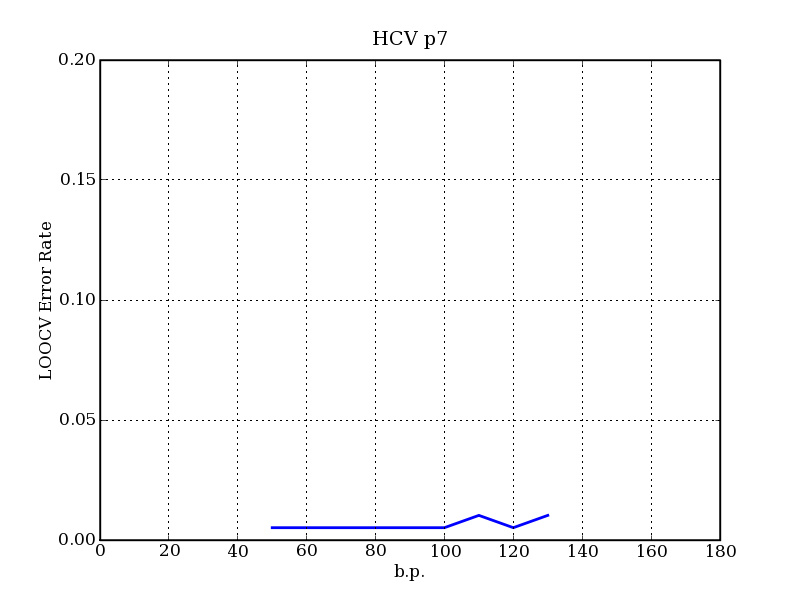


Figure 3(e)


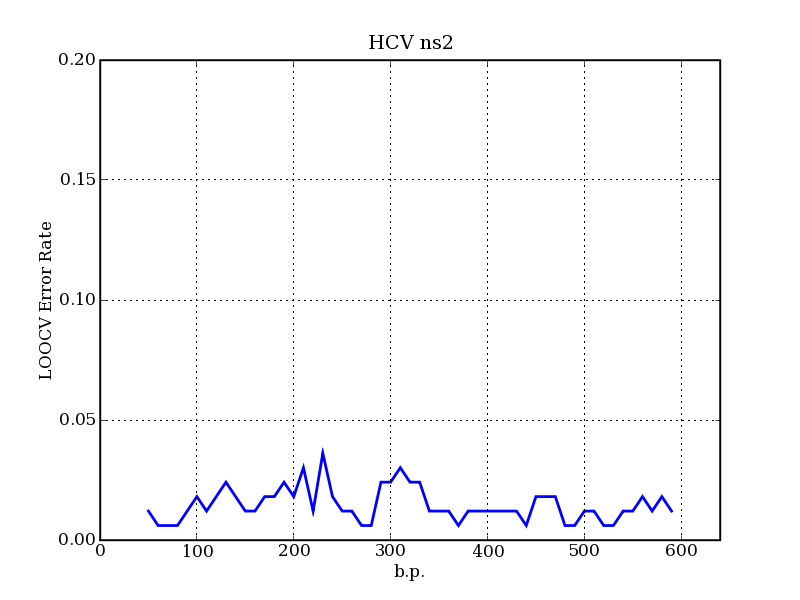


Figure 3(f)


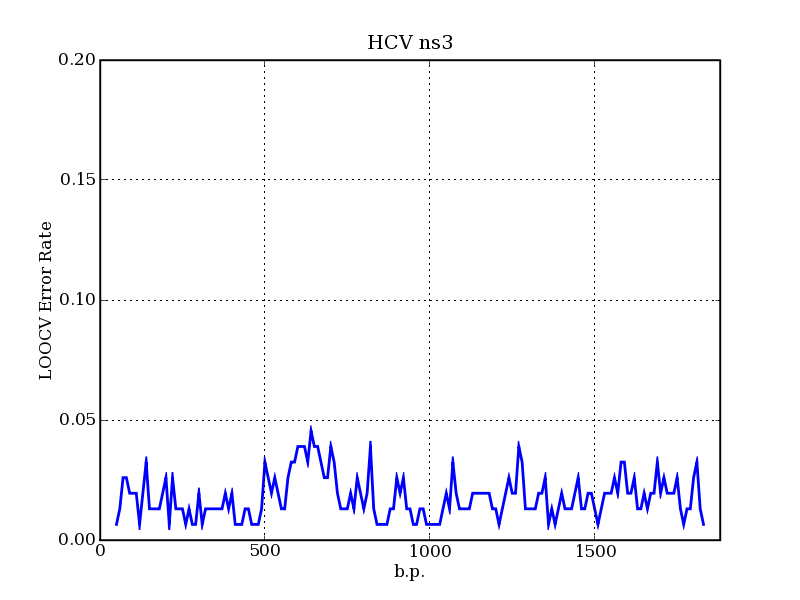


Figure 3(g)


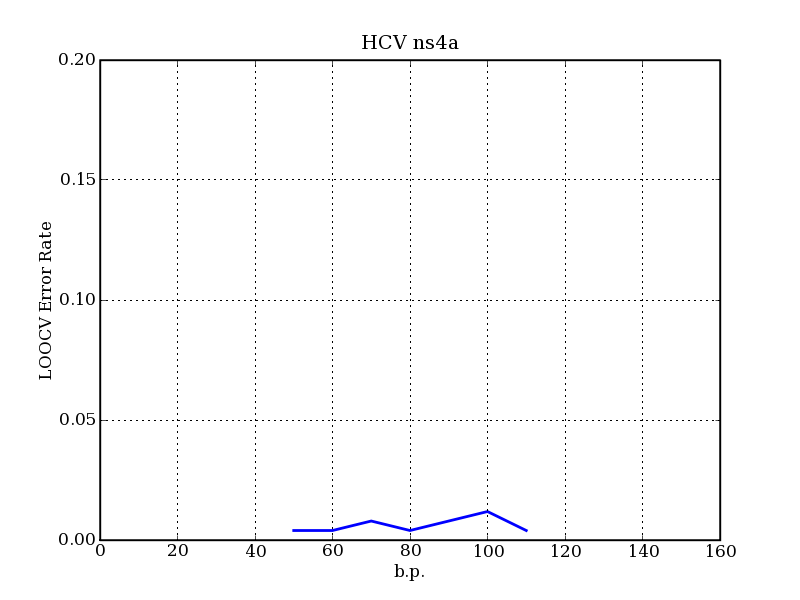


Figure 3(h)


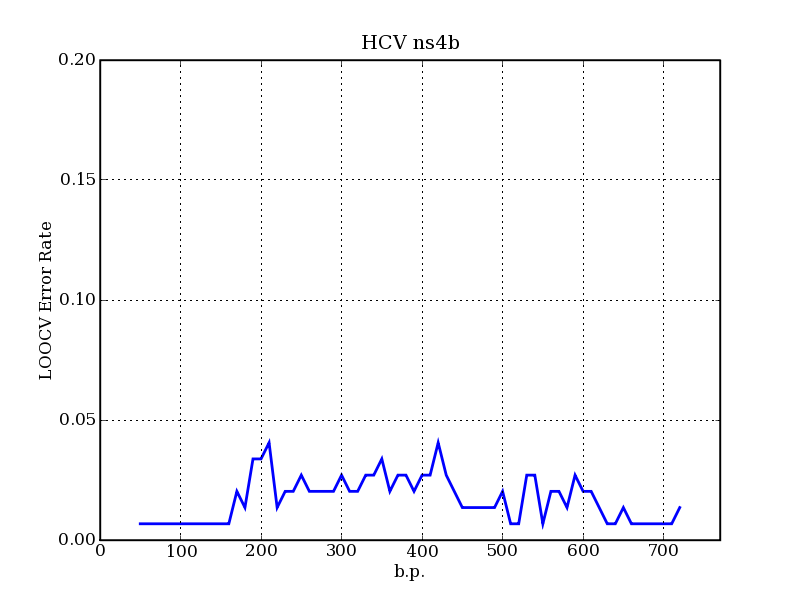


Figure 3(i)


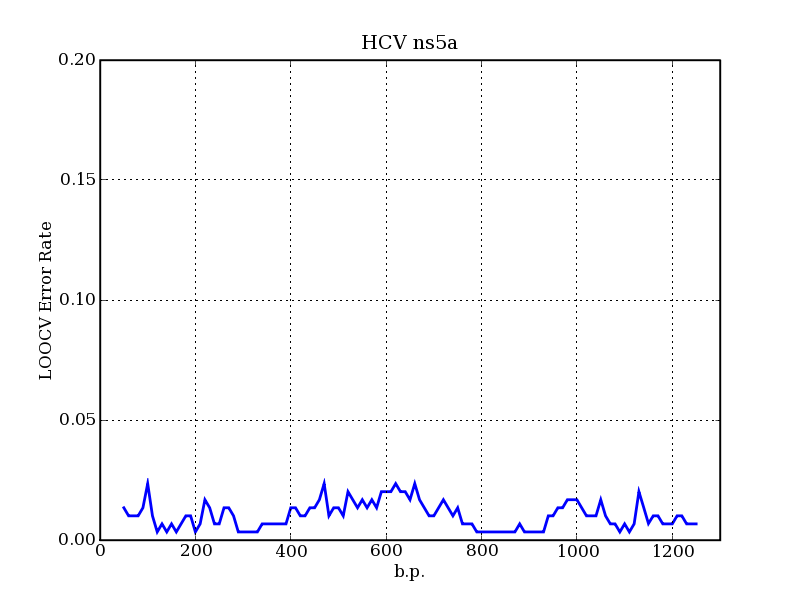


Figure 3(j)


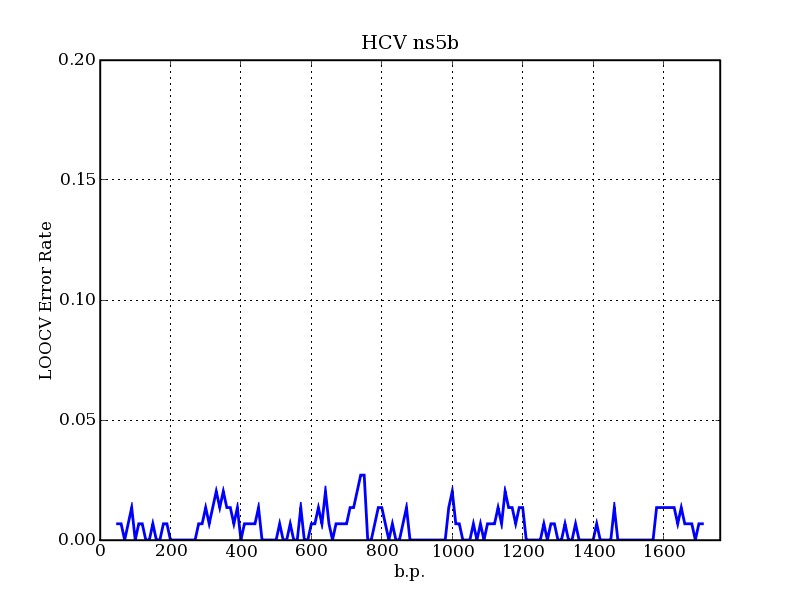


Figure 3(k)


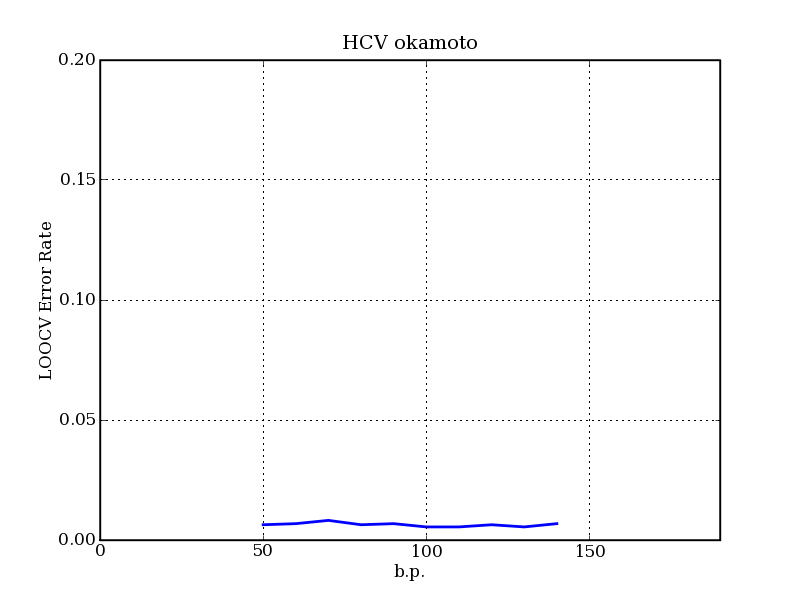


Figure 3(l)

Figure 4(a)

Figure 4(b)


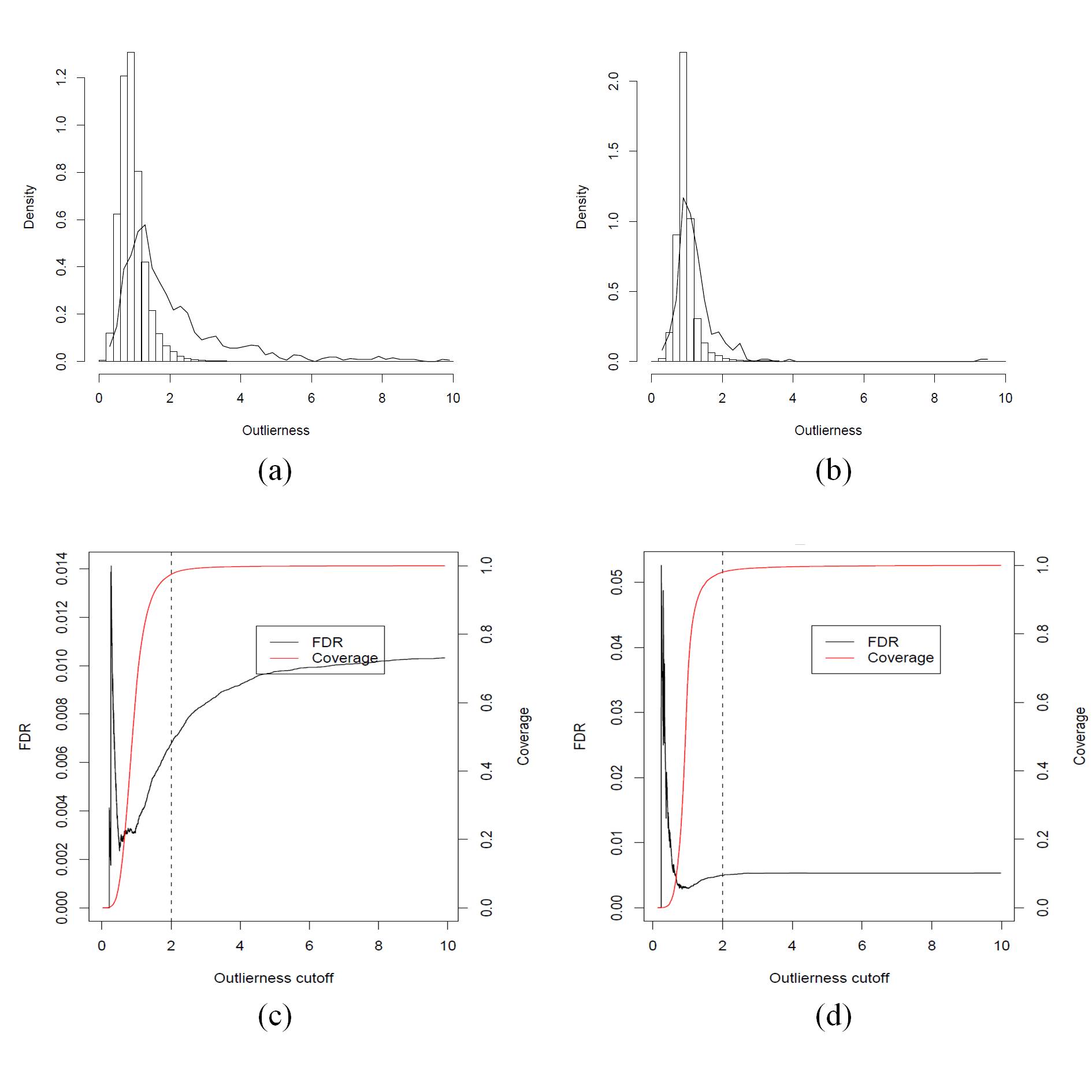


Figure 5

Figure 6(a) Figure 6(b)

Figure 6(c)
